# Supplementary material for: Targeting histone H2B acetylated enhanceosomes via p300/CBP degradation in prostate cancer
Source: Nat Genet. 2025 Oct 3;57(10):2468–81. doi: 10.1038/s41588-025-02336-6 (PMC12513837; doi:10.1038/s41588-025-02336-6)
Supplement: Supplementary file 1 — Supplementary Methods. [file 41588_2025_2336_MOESM1_ESM.pdf]

nature genetics

Supplementary information https://doi.org/10.1038/s41588-025-02336-6

Targeting histone H2B acetylated enhanceosomes via p300/CBP degradation in prostate cancer

In the format provided by the authors and unedited

Supplementary Notes

Supplementary Methods:

RNA extraction and quantitative polymerase chain reaction

Total RNA was extracted from cells using QIAzol Lysis Reagent (QIAGEN). Initially, $1x10^6$ cells were lysed with 700 µL of QIAzol. The lysates were incubated at room temperature for 5 minutes to dissociate nucleoproteins, then mixed with 140 µL chloroform and centrifuged at 12,000 g for 15 minutes at 4°C to achieve phase separation. The aqueous phase was then mixed with 1.5x volume of ethanol and applied to a RNeasy Mini spin column (QIAGEN) for RNA purification, following the miRNeasy Mini Kit protocol. The RNA was then eluted in RNase-free water, and its concentration and purity assessed using a NanoDrop spectrophotometer.

For quantitative PCR (qPCR) analysis, cDNA was synthesized using the extracted RNA as a template. Reverse transcription was carried out using Maxima First Strand cDNA Synthesis Kit (ThermoFisher), following the manufacturer's instructions. The resulting cDNA was then used for qPCR using SYBR™ Green PCR Master Mix (Applied Biosystems), depending on the specific gene targets. The qPCR reactions were performed in QuantStudio 5 Real-Time PCR system (Applied Biosystems), and the data were analyzed using the ΔΔCt method to quantify gene expression levels, normalizing to the expression of the *GAPDH* gene. Primers used in this study are listed in **Supplementary Table 2**.

CRISPR knockout

Short guide RNAs (sgRNAs) targeting human p300 or CREB-binding protein (CBP) exons were designed using Benchling (https://www.benchling.com/) and cloned into the lentiCRISPR v2 plasmid, as previously described[1]. LNCaP and 22Rv1 cells were transiently transfected with

lentiCRISPR v2 vectors encoding either non-targeting control or pairs of independent sgRNAs for p300 or CBP. Forty-eight hours post-transfection, cells were selected with 1 μg/mL puromycin for three days. Knockout efficiency was assessed by Western blot. Primers used in this study are listed in **Supplementary Table 2**.

siRNA-mediated gene knockdown

The human non-targeting control (Cat#: D-001810-10-05), p300 (Cat#: L-003486-00-0005), and CBP (Cat#: L-003477-00-0005) ON-TARGETplus SMARTPool siRNAs were ordered from Horizon Discovery. Cells were plated in a 6-well plate at the density of 300,000 cells per well. After 24 hours, cells were transfected with 30 nM of siRNAs using the RNAiMAX transfection reagents (Life Technologies) on two consecutive days. The protein was extracted on day 3 to identify efficient (>80%) knockdown of the target genes.

Acetyl (lysine)-proteomics analysis

Sample preparation:

Frozen cell pellets were lysed in 8 M urea buffer (100 mM Tris-HCl, pH 8.0, with 1% protease inhibitor) by sonication. After centrifugation (12,000 ×g, 15 min, 4 °C), protein concentrations were determined using the BCA assay. Two milligrams of protein per sample were reduced with 4.5 mM DTT at 37 °C for 1 hour and alkylated with 10 mM iodoacetamide at room temperature for 30 minutes in the dark. Samples were diluted with 50 mM ammonium bicarbonate and digested overnight at 37 °C with trypsin (1:200 enzyme-to-protein ratio). Digestion was stopped with 1% TFA, and peptides were desalted using C18 columns and eluted with 50% acetonitrile in 0.1% TFA. For acetylated peptide enrichment, desalted peptides were resuspended in IAP buffer and

incubated with anti-acetyl-lysine antibody beads (Cell Signaling Technology) at 4 °C for 4 hours. Beads were washed, and acetylated peptides were eluted with 0.1% TFA and dried.

Liquid chromatography-mass spectrometry (LC-MS)/MS:

Peptides were analyzed using an Ultimate 3000 nano ultra high-performance liquid chromatography (UHPLC) system coupled to a Q Exactive HF mass spectrometer with an ESI nanospray source (Thermo Fisher Scientific). Peptides were separated on a PepMap C18 column (75 µm × 50 cm) using a linear gradient of 2–40% buffer B (0.1% formic acid in 80% acetonitrile) over 120 minutes at a flow rate of 250 nL/min. MS1 scans were acquired from 300–1,650 m/z at 60,000 resolution. MS/MS was performed in Top20 mode with HCD fragmentation (NCE 28%), using 15,000 resolution for MS2 scans. Dynamic exclusion was set to 30 seconds, and singly charged or unassigned precursor ions were excluded.

Data analysis:

Raw MS files were analyzed and searched against the Homo sapiens protein database based on the species of the samples using Maxquant (2.3.0.0). The parameters were set as follows: the protein modifications were Carbamidomethylation (C), Oxidation (M) (variables), Acetyl (K) (variables), Acetyl (Protein N-term) (variables), Acetyl (N-term) (variables); the enzyme specificity was set to trypsin; the maximum missed cleavages were set to 5; the precursor ion mass tolerance was set to 10 ppm, and MS/MS tolerance was 0.5 Da.

Nascent RNA-seq data analysis

Nascent RNA sequencing was conducted using the Click-iT™ Nascent RNA Capture Kit

(Invitrogen). Cells were treated with or without CBPD-409 for 2 and 4 hours, with 0.5 mM 5-Ethynyl Uridine (EU) added to the medium 45 minutes before cell harvest. After treatment, cells were washed with phosphate buffered saline (PBS), harvested, and lysed with the kit's lysis buffer. RNA isolation was performed using QIAzol reagent and RNeasy Mini spin column (Qiagen), following previously described methods[1]. For ribosomal RNA depletion, 5 µg of total RNA were processed using the Ribominus™ Eukaryote System v2 (ThermoFisher). The rRNA-depleted, EU-labeled RNA (250-500 ng) was then incubated with 0.25 mM biotin azide in the Click-iT® reaction cocktail for 30 minutes with gentle vortexing. Biotin-conjugated EU-RNA was precipitated using UltraPure™ Glycogen (1 µL, ThermoFisher), 7.5 M ammonium acetate (50 µL, Sigma-Aldrich), and chilled 100% ethanol (700 µL) at –70°C overnight. The sample was then centrifuged at 13,000 × g for 20 minutes at 4°C, and the RNA pellet was washed with 75% ethanol. Finally, 100-200 ng of the purified EU-RNA was used for library preparation and sequencing, as detailed in the RNA-seq section.

The sequencing reads were mapped to the reference genome (GRCh38.p14) downloaded from Gencode[2] using Burrows-Wheeler Alignment Tool (bwa mem) with default parameters[3]. The resulting sam files were then converted to bam format and sorted using Samtools[4]; bigwig files were then generated using bamCoverage of deeptools[5] with reads per kilobase per million mapped reads (RPKM) normalization method. Quantification of gene and enhancer expression levels was conducted with FeatureCounts[6]. Androgen receptor (AR) binding sites at non-promoter regions were defined as AR enhancers. The coordinates of AR enhancers were based on chromatin immunoprecipitation sequencing (ChIP-seq) peaks of AR, and peaks that overlap with gene promoters (+/- 3 kb of transcription start site (TSS)) were excluded. Normalized enhancer expression levels in transcripts per million (TPM) were used for data visualization. Meta profile

plots were generated with the plotProfile function of deeptools[5], in which the inputs were merged bigwig files from duplicate libraries using University of California Santa Cruz (UCSC) BigwigMerge tool[7].

Enrichment heatmaps and profile plots

The read density heatmaps and enrichment plots were all created using the software Deeptools. The referencePoint parameter was set to +/- 2.5 kb for histone signals and +/- 1 kb for other signals. Other settings included using 'skipzeros', 'averagetype mean', and 'plotype se'. The Encode blacklist ENCFF356LFX was used. Non-promoter regions were selected based on annotation by R package ChIPseeker[8] with +/- 1kb windows to gene regions.

Peak annotations and overlaps

The R package ChIPpeakAnno[9] was used to compare samples' peak lists from MACS[10]. Peaks were reduced within 500 bp, and overlaps were calculated using settings maxgap=-1L, minoverlap=0L, ignore.strand=TRUE, connectedPeaks=c('keepAll', 'min', 'merge'). An additional R package, ChIPseeker[8], was used for comparisons of the enrichment sites to the known gene database (TxDb.Hsapiens. UCSC.hg38.knownGene) with a +/- 1kb relative distance from gene regions.

Analysis of AR binding sites from human castration-resistant prostate cancer (CRPC) and benign prostate tissues

Additional published datasets from Pomerantz et al.[11,12] were processed using our ChIP-seq pipeline. Aligned BAM files were merged using Picard's MergeSamFiles, and downsampled to

~100 million reads for AR datasets using samtools view -bs. Peaks were re-called with MACS2 callpeak, and output bedgraphs were converted to bigWig format using wigToBigWig.

ChIP-seq peaks quartile categorization and heatmaps

Quartile comparisons were made by taking results from MACS peak calling into R as granges objects and labeled into quartile bins by MACS score. Overlap analysis was performed by IRanges's subsetbyoverlaps[13]. After some table manipulation and calculations for percentile in bash, data was reloaded into R and plotted as barplots with ggplot.

HiChIP-seq data analysis

HiChiP-seq data from VCaP DMSO histone H3 lysine 4 trimethylation (H3K4me3) (GSM5229035) was used to gain information about interactions linked to the peaks called by ChIP-Seq in our generated datasets. Bedtools was used to compare each end of the loop information from HiChiP-seq dataset to the peaks classified as either AR only or shared between AR and P300. The opposite end of each matched loop was then taken and filtered by annotation with ChipPeakAnno, only promoters were kept. Genes associated with each were used to filter the EU nascent TPM counts, with name conversion by biomart R package. Ggplot2 was then used to plot the EU data as a violin plot.

Boyden chamber invasion assay

LNCaP and 22RV1 cells were grown in 10% charcoal-stripped fetal bovine serum (FBS) medium for 24 h. $3x10^5$ starved LNCaP cells or $5x10^4$ 22RV1 cells were resuspended in 500 µL serum-free RPMI-1640 medium with addition of 100 nM CBPD-409, GNE-049, or CCS1477 and were added

to Matrigel coated invasion chambers (Corning, 354480). 750 µL of 20% FBS RPMI-1640 medium was added to the bottom wells. After 36 h, the invasion chambers were fixed by 100% methanol for 10 minutes and stained by crystal violet for 10 minutes. The images were taken by EVOS Cell Imaging Systems from Thermo Fisher.

Cohort generation

Two cohort systems were generated for the in-situ validation. The initial step involved analyzing a complete tissue section cohort to assess the expression levels of p300, CBP, histone H2B lysine 5 acetylation (H2BK5ac), and histone H2B lysine 20 acetylation (H2BK20ac) in relation to Keratin8 (KRT8). This cohort consisted of 10 primary prostate cancer tissues, along with matched normal adjacent tissues from 9 patients. To examine the co-expression of p300 with histone H2B N-terminus acetylation (H2BNTac: H2BK5ac/H2BK20ac) and CBP with H2BNTac (H2BK5ac/H2BK20ac), we collected two in-house progressive prostate cancer tissue microarrays (TMAs), identified as TMA-145 and TMA-200. These TMAs consist of 156 cores, including 84 cores from benign tissue and 72 from prostatic adenocarcinoma. The samples were obtained from 56 patients, which included 27 patients with primary prostate cancer (with 28 samples) and 29 patients with benign prostatic tissue (with 35 samples). The downstream pathological evaluation of multi-plex immunofluorescence staining was performed with the help of Fiji (v1.54f), formerly Image J (details below). Further cohort details are available from **Supplementary Table 3**.

Imaging and analysis

The multiplex immunofluorescence slides were imaged with LSM 900 upright confocal microscope with Airyscan mode. Each tissue was acquired as a merged channel image and individual single channel images. Quantification of IF signal was performed using Fiji (v1.54f). The Integrated Optical Density (IOD) was calculated using the "Measure" function within the regions of interest (ROIs) in each individual channel image. For the matched cohort, ROIs were generated based on KRT8 IF staining using Cellpose v2 (model: "Cyto2). For the TMAs, the nuclear ROIs were generated based on DAPI staining using native "Threshold" function with "Otsu" method and then segmentation of each nucleus was performed using "Analyze Particles" function. The Integrated Optical Density (IOD) was calculated using the "Measure" function within the regions of interest (ROIs) in each individual channel image. Plots displaying the results were generated using ggplot2 (v3.5.1) package in R (v4.4.1).

CBPD-409 and enzalutamide formula for *in vivo* studies

CBPD-409 was freshly dissolved in 100% PEG400 before administration to mice. Enzalutamide was prepared in 1% carboxymethyl cellulose (CMC) with 0.25% Tween-80 and homogenized by sonication. Both CBPD-409 and enzalutamide were administered to mice by oral gavage.

Prostate patient-derived xenograft models

The patient-derived xenograft (PDX) MDA-PCa-146-12 model was obtained from the University of Texas M.D. Anderson Cancer Center. This AR-positive PDX was developed from a CRPC patient as previously described[14].

The prostate cancer sample used for WA74 PDX development was collected during a rapid autopsy case as part of the Michigan Legacy Tissue Program (MTLP). For this line, metastases

were excised from the intestinal mesentery and immediately placed into cold DMEM. Within 2-3 hours, tumor chunks were implanted into subcutaneous pockets of male NOD scid gamma (NSG) mice. Mice were monitored weekly. Out of 5 mice, only one animal showed tumor growth after 10 months. This tumor was harvested and sequentially passaged in both NSG and CB17SCID mice. This WA74 PDX line bears *BRCA2* somatic mutation and *TMPRSS2-ERG* fusion.

For the intact WA74 model, the PDXs were propagated in male severe combined immunodeficient mice (SCID) mice. This involved surgical implantation of 2 mm^3 tumor pieces, encapsulated in 100% Matrigel, into the flanks of the mice. After the tumor grew to 200 mm^3, mice were randomized into different treatment groups. These groups were administered with vehicle, 10 mg/kg enzalutamide (5 times per week), or 3 mg/kg CBPD-409 (3 times per week) with 10 mg/kg enzalutamide by oral gavage for 4 weeks.

For CRPC MDA-PCa-146-12 and CRPC WA-74 models, tumors were established in castrated male SCID mice. When tumor sizes reached about 100 mm^3, similar randomization and division into treatment groups were conducted. These groups received the same treatments as intact WA-74 tumors for 4-5 weeks.

Throughout all studies, the treatment protocols strictly followed the Institutional Animal Care and Use Committee (IACUC) guidelines, ensuring that the maximum tumor size did not surpass 2.0 cm in any dimension. Mice with xenografts reaching this threshold were humanely euthanized.

Drug toxicity analysis in CD-1 mice, *Crbn*$^{V380E/I391V}$ C57BL/6 mice, and CD rats

Complete blood cell count:

Blood samples were collected from mice or rats using the submandibular bleeding method,

employing K3 EDTA tubes to prevent coagulation. Each animal was gently restrained, and approximately 100-200 µL of blood was carefully obtained from the cheek pouch, ensuring minimal discomfort to the animal. This method was chosen for its minimal invasiveness and reliability in obtaining sufficient blood volume for analysis. Following collection, the blood samples were immediately mixed with EDTA by gentle inversion to prevent clotting. The complete blood cell count (CBC) was performed on the same day as the blood collection to maintain sample integrity. Samples were analyzed by the University of Michigan *in vivo* animal core (IVAC) using a calibrated hematology analyzer suitable for mouse blood. WBC: White Blood Cell; NEU: Neutrophils; LYM: Lymphocytes; MONO: Monocytes; EOS: Eosinophils; BAS: Basophils; NEU%: Neutrophils (percentage of total WBC); LYM%: Lymphocytes (percentage of total WBC); MONO%: Monocytes (percentage of total WBC); EOS%: Eosinophils (percentage of total WBC); BAS%: Basophils (percentage of total WBC); RBC: Red Blood Cell; HGB: Hemoglobin; HCT: Hematocrit; MCV: Mean Corpuscular Volume; MCH: Mean Corpuscular Hemoglobin; MCHC: Mean Corpuscular Hemoglobin Concentration; RDW%: Red Cell Distribution Width (percentage); PLT: Platelet count; MPV: Mean Platelet Volume.

Serum chemistry analysis:

Serum was obtained for biochemical analysis through a standardized collection and separation process. More than 200 µL of blood was drawn into serum separator tubes, and tubes were set aside to allow the blood to clot for over 30 minutes. Following the clotting period, the tubes were centrifuged at 1800-3000g for 10 minutes. The cell-free serum was then extracted from the tubes for analysis at IVAC. ALT: Alanine Aminotransferase; BUN: Blood Urea Nitrogen; TBILI: Total Bilirubin; ALB: Albumin; CREA: Creatinine.

Histopathological analysis of organs:

Multiple organs including the liver, spleen, kidney, colon, small intestine, mesenteric lymph nodes, pancreas, prostate, and testis were systematically evaluated for histopathological changes as previously described[1]. Two pathologists, blinded to the control and treatment groups, examined the stained sections under a brightfield microscope. They assessed general tissue morphology and architectural coherence across all organs. Detailed analyses were conducted at cellular and sub-cellular levels, focusing on specific aspects unique to each organ. Johnsen scoring was done according to the well-established schema detailed in published literature[15,16].

Alcian blue staining:

Alcian blue staining was performed using the Alcian Blue Stain Kit (pH 2.5, Abcam) following the manufacturer's protocol. Tissue sections on slides were first incubated overnight at 58°C, then deparaffinized with xylene and rehydrated through a series of ethanol solutions (100%, 70%) and water, each for 5 minutes. The slides were then treated with acetic acid solution for 3 minutes, followed by incubation in Alcian blue stain (pH 2.5) for 30 minutes at room temperature. After staining, the slides were rinsed in acetic acid for 1 minute and washed three times with water, 2 minutes each. Nuclear Fast Red was applied as a counterstain for 5 minutes, followed by washing, dehydration in ethanol and xylene, and mounting with EcoMount (Thermo Fisher).

The Alcian blue goblet cell: epithelial ratio (GC:EC) was calculated by recording the number of goblet cells and epithelial cells per colonic crypt. In total, 10 colonic crypts were assessed per mouse sample, and the final average score was rendered.

Analysis of drug synergism

To evaluate potential synergistic interactions between two pharmacological agents, cells were subjected to escalating concentrations of each drug individually over a period of 5 days. The assessment of cell viability post-treatment was conducted using the CellTiter-Glo Luminescent Cell Viability Assay (Promega). This experimental procedure was replicated across four biological replicates. The resulting data were analyzed to quantify the percentage of inhibition relative to untreated control cells. To determine the presence and extent of drug synergy, the data were processed using the online version of the Synergy Finder tool. This analysis employed the Bliss independence model to interpret interactive effects between the two drugs.

Synthetic Chemistry for CBPD-409 and CBPD-409-Me

1. General chemistry information

All commercial materials were utilized in their original form, unless explicitly stated otherwise. NMR spectra were captured using a Bruker Ascend™ 400 MHz spectrometer, with calibration conducted using residual solvent peaks as internal references. Spectral data were presented in the (δ) chemical shift (multiplicity, J values in Hz, integration) format, employing abbreviations such as s = singlet, d = doublet, t = triplet, q = quartet, hept = heptet, dd = doublet of doublets, and m = multiplet. Low-resolution mass spectrometry (MS) analysis was performed using a Waters UPLC ACQUITY QDa mass spectrometer. High-resolution mass experiments were executed on an Agilent Technologies 6230 TOF LC/MS instrument with APCI ionization. Flash column chromatography was undertaken with a Teledyne CombiFlash RF+ using RediSep Rf silica gel flash columns. The final compounds and some intermediates underwent purification using a C18 reversed-phase preparative HPLC column (SunFire™ Prep C18 OBD™ 5 µm,

50×100 mm) with solvent A (0.1% TFA in H_2O) and solvent B (0.1% TFA in MeCN) as eluents at a flow rate of 60 mL/min. The purity of all final compounds was evaluated through UPLC-MS analysis (10-100% MeCN in H_2O containing 0.1% formic acid in 5 min, 1.0 mL/min flow rate) with a C18 column (ACQUITY UPLC BEH C18 1.7 µm, 2.1 × 50 mm).

Abbreviations used: $CDCl_3$, deuterated chloroform; Cs_2CO_3, cesium carbonate; DCM, dichloromethane; DMAP, 4-dimethylaminopyridine; DMF, *N,N'*-Dimethylformamide; DMSO, dimethyl sulfoxide; DIBAL, diisobutylaluminum hydride; DIPEA, *N,N'*-Diisopropylethylamine; Et_3N, triethylamine; EtOAc, ethyl acetate; HCl, hydrochloric acid; K_2CO_3, potassium carbonate; KOH, potassium hydroxide; MeCN, acetonitrile; MeI, methyl iodide; MeOH, methanol; N_2, nitrogen; Na_2SO_4, sodium sulfate; $NaBH(OAc)_3$, sodium triacetoxyborohydride; NaH, sodium hydride; NaOH, sodium hydroxide; NBS, N-bromosuccinimide; $PdCl_2$(dppf), palladium(II) chloride-bis(diphenylphosphino)ferrocene; RuPhos, ruthenium-based ligand (Phospha-methyl-di(tert-butyl)phenylphosphine); RuPhos Pd G2, second-generation ruthenium-phosphine ligand; *t*-BuONa, sodium tert-butoxide; TFA, trifluoroacetic acid; THF, tetrahydrofuran.

2. Method and procedure for the preparation of CBPD-409

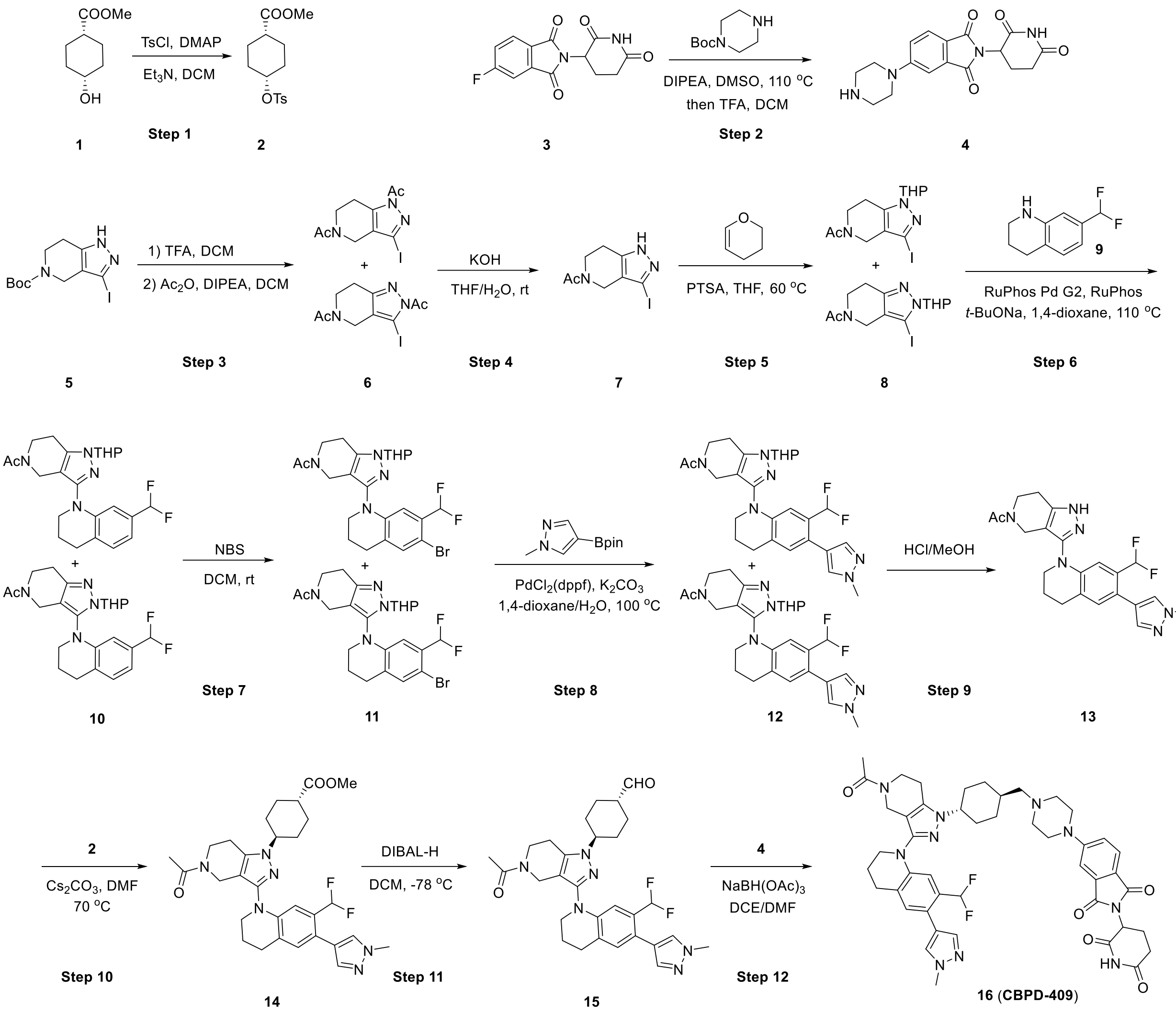

Step 1: Synthesis of *cis*-4-(tosyloxy)cyclohexane-1-carboxylate (**2**)

To a solution of methyl *cis*-4-hydroxycyclohexane-1-carboxylate (**1**, 5 g, 1.0 eq) in DCM (50 mL), 4-methylbenzenesulfonyl chloride (9.0 g, 1.5 eq), DMAP (0.77 g, 0.2 eq) and Et_3N (13.2 mL, 3.0 eq) were sequentially added. The resulting mixture was stirred at room temperature for 16 h. Following this, water was introduced, and the resulting mixture was subjected to extraction with DCM. The combined organic layer was then washed with brine, dried over anhydrous Na_2SO_4, filtered, and concentrated under reduced pressure. The resulting crude residue was subsequently subjected to purification through flash column chromatography (20% EtOAc/*n*-hexane) to give

cis-4-(tosyloxy)cyclohexane-1-carboxylate (**2**) as a yellow oil (9.3 g, 94% yield). LC-MS: *m/z* $[M+H]^+$ 334.85; ^{1}H NMR (400 MHz, Chloroform-*d*) δ 7.81 – 7.75 (m, 2H), 7.35 – 7.29 (m, 2H), 4.70 (tt, J = 5.0, 2.9 Hz, 1H), 3.66 (s, 3H), 2.44 (s, 3H), 2.37 – 2.26 (m, 1H), 1.92 – 1.78 (m, 4H), 1.75 – 1.65 (m, 2H), 1.59 – 1.47 (m, 2H).

Step 2: Synthesis of 2-(2,6-dioxopiperidin-3-yl)-5-(piperazin-1-yl)isoindoline-1,3-dione (**4**)

To a mixture of 2-(2,6-dioxopiperidin-3-yl)-5-fluoroisoindoline-1,3-dione (**3**, 570 mg, 1.0 eq) and *tert*-butyl piperazine-1-carboxylate (769 mg, 2.0 eq) in DMSO (10 mL) was added DIPEA (1.44 mL). Upon cooling to room temperature, the reaction mixture was diluted with DCM, followed by washing with brine, drying over anhydrous Na_2SO_4, and concentration under reduced pressure. The resulting residue underwent purification via flash column chromatography (using a gradient of 0-5% MeOH/DCM) to yield a pure product, which was then subjected to treatment with TFA, resulting in the formation of 2-(2,6-dioxopiperidin-3-yl)-5-(piperazin-1-yl)isoindoline-1,3-dione (**4**) as a light yellow solid (850 mg, yield = 90%). UPLC-MS [M+H]+ 343.15; ^{1}H NMR (400 MHz, MeOH-d_4) δ 7.77 (d, *J* = 8.5 Hz, 1H), 7.48 (d, *J* = 2.3 Hz, 1H), 7.35 (dd, *J* = 8.5, 2.4 Hz, 1H), 5.10 (dd, *J* = 12.5, 5.5 Hz, 1H), 3.76 – 3.65 (m, 4H), 3.43 – 3.37 (m, 4H), 2.93 – 2.81 (m, 1H), 2.80 – 2.66 (m, 2H), 2.16 – 2.08 (m, 1H).

Step 3: Synthesis of 1,1'-(3-iodo-6,7-dihydro-1H-pyrazolo[4,3-c]pyridine-1,5(4H)-diyl)bis(ethan-1-one) and 1,1'-(3-iodo-6,7-dihydro-2H-pyrazolo[4,3-c]pyridine-2,5(4H)-diyl)bis(ethan-1-one) (**6**)

A solution of *tert*-butyl 3-iodo-1,4,6,7-tetrahydro-5H-pyrazolo[4,3-c]pyridine-5-carboxylate (**5**, 2.5 g, 1.0 eq) in DCM (30 mL) was treated with TFA (10 mL). The reaction mixture was stirred at room temperature for 30 minutes, after which it was concentrated under reduced pressure. The resulting residue was dissolved in DCM (30 mL), and the solution was cooled to

0 °C. DIPEA (6.2 mL, 5.0 eq) was then added, followed by acetic anhydride (1.8 mL, 2.5 eq). The reaction was allowed to warm to room temperature and was stirred for 20 h. After completion, the reaction mixture was diluted with DCM, washed with brine, dried over anhydrous Na_2SO_4, and concentrated under reduced pressure to remove the solvent. The resulting crude product was subjected to purification via flash column chromatography (using a gradient of 0-5% MeOH/DCM) to afford 1,1'-(3-iodo-6,7-dihydro-1H-pyrazolo[4,3-c]pyridine-1,5(4H)-diyl)bis(ethan-1-one) and 1,1'-(3-iodo-6,7-dihydro-2H-pyrazolo[4,3-c]pyridine-2,5(4H)-diyl)bis(ethan-1-one) (**6**) as a white solid (2.2 g) in a yield of 92%. LC-MS *m/z* $[M+H]^+$ 334.04; ^1H NMR (400 MHz, Chloroform-*d*) δ 4.46 – 4.22 (m, 2H), 3.88 – 3.65 (m, 2H), 3.20 – 3.07 (m, 2H), 2.67 (s, 3H), 2.19 (s, 3H).

Step 4: Synthesis of 1-(3-iodo-1,4,6,7-tetrahydro-5H-pyrazolo[4,3-c]pyridin-5-yl)ethan-1-one (**7**)

A solution of compound **6** (3.4 g, 1.0 eq) in THF/H_2O (40 mL/20 mL) was treated with KOH (2.9 g, 5.0 eq). The reaction mixture was stirred at room temperature for 3 h. Following this, the solvent was removed under reduced pressure, and the resulting residue was dissolved in water. The solution was then neutralized with 2 N aqueous HCl to achieve a pH of 8~9. The neutralized solution was subsequently extracted with EtOAc. The combined organic layers were washed with brine, dried over anhydrous Na_2SO_4, and concentrated to yield 1-(3-iodo-1,4,6,7-tetrahydro-5H-pyrazolo[4,3-c]pyridin-5-yl)ethan-1-one (**7**) as a white solid (2.7 g), with a yield of 91%. LC-MS *m/z* $[M+H]^+$ 291.89; ^1H NMR (400 MHz, Methanol-d_4) δ 12.97 (s, 1H), 4.22 (s, 2H), 3.76 – 3.60 (m, 2H), 2.77 – 2.54 (m, 2H), 2.13 – 2.05 (m, 3H).

Step 5: Synthesis of 1-(3-iodo-1-(tetrahydro-2H-pyran-2-yl)-1,4,6,7-tetrahydro-5H-pyrazolo[4,3-c]pyridin-5-yl)ethan-1-one and 1-(3-iodo-2-(tetrahydro-2H-pyran-2-yl)-2,4,6,7-tetrahydro-5H-pyrazolo[4,3-c]pyridin-5-yl)ethan-1-one (**8**)

In a solution containing compound **7** (3.7 g, 1.0 eq) and 3,4-dihydro-2*H*-pyran (3.5 mL, 3.0 eq) in THF (60 mL), *p*-toluenesulfonic acid (2.2 g, 1.0 eq) was added. The reaction mixture was stirred at 65 °C for 24 h. After completion, the reaction mixture was concentrated, then diluted with DCM. The resulting solution was washed with brine, dried over anhydrous Na_2SO_4, and concentrated under reduced pressure to remove the solvent. The residue was subjected to purification by flash column chromatography (using a gradient of 0-100% EtOAc/*n*-hexane) to afford 1-(3-iodo-1-(tetrahydro-2H-pyran-2-yl)-1,4,6,7-tetrahydro-5H-pyrazolo[4,3-c]pyridin-5-yl)ethan-1-one and 1-(3-iodo-2-(tetrahydro-2H-pyran-2-yl)-2,4,6,7-tetrahydro-5H-pyrazolo[4,3-c]pyridin-5-yl)ethan-1-one (**8**) as a white solid (3.6 g, 76% yield). LC-MS *m/z* $[M+H]^+$ 376.14; ^1H NMR (400 MHz, DMSO-d_6) δ 5.33 (dd, J = 9.7, 2.6 Hz, 1H), 4.34 – 4.09 (m, 2H), 3.89 – 3.81 (m, 1H), 3.80 – 3.53 (m, 3H), 2.91 – 2.55 (m, 2H), 2.23 – 2.04 (m, 4H), 2.01 – 1.90 (m, 1H), 1.89 – 1.78 (m, 1H), 1.71 – 1.57 (m, 1H), 1.57 – 1.44 (m, 2H).

Step 6: Synthesis of 1-(3-(7-(difluoromethyl)-3,4-dihydroquinolin-1(2H)-yl)-1-(tetrahydro-2H-pyran-2-yl)-1,4,6,7-tetrahydro-5H-pyrazolo[4,3-c]pyridin-5-yl)ethan-1-one and 1-(3-(7-(difluoromethyl)-3,4-dihydroquinolin-1(2H)-yl)-2-(tetrahydro-2H-pyran-2-yl)-2,4,6,7-tetrahydro-5H-pyrazolo[4,3-c]pyridin-5-yl)ethan-1-one (**10**)

A mixture of intermediate **8** (2.0 g, 1.0 eq), 7-(difluoromethyl)-1,2,3,4-tetrahydroquinoline (**9**, 1.4 g, 1.2 eq), RuPhos Pd G2 (828 mg, 0.2 eq), RuPhos (500 mg, 0.2 eq), and *t*-BuONa (2.3 g, 4.5 eq) in dioxane (40 mL) was degassed and purged with nitrogen three times. The mixture was then stirred at 110 °C for 12 h. LC-MS analysis indicated complete consumption of **8**, with the formation of a main peak exhibiting the desired mass spectrum. Upon cooling, the mixture was diluted with DCM, filtered through Celite, and the filter cake was washed with DCM. The filtrate was concentrated under reduced pressure, and the resulting residue was purified by flash column

chromatography (using a gradient of 0-100% EtOAc/*n*-hexane) to afford 1-(3-(7-(difluoromethyl)-3,4-dihydroquinolin-1(2H)-yl)-1-(tetrahydro-2H-pyran-2-yl)-1,4,6,7-tetrahydro-5H-pyrazolo[4,3-c]pyridin-5-yl)ethan-1-one and 1-(3-(7-(difluoromethyl)-3,4-dihydroquinolin-1(2H)-yl)-2-(tetrahydro-2H-pyran-2-yl)-2,4,6,7-tetrahydro-5H-pyrazolo[4,3-c]pyridin-5-yl)ethan-1-one (**10**, 1.7 g) as a light yellow foam in a yield of 74%. LC-MS *m/z* $[M+H]^+$ 431.28; ^{1}H NMR (400 MHz, Chloroform-*d*) δ 7.12 – 7.00 (m, 1H), 6.84 – 6.74 (m, 1H), 6.67 (s, 1H), 6.61 – 6.28 (m, 1H), 5.26 – 5.16 (m, 1H), 4.26 – 4.13 (m, 1H), 4.09 – 3.98 (m, 2H), 3.94 – 3.81 (m, 1H), 3.77 – 3.59 (m, 4H), 2.94 – 2.75 (m, 4H), 2.41 – 2.28 (m, 1H), 2.17 – 1.92 (m, 7H), 1.70 – 1.56 (m, 3H); ^{13}C NMR (101 MHz, $CDCl_3$) δ 169.79, 169.13, 149.54, 149.01, 143.00, 138.94, 137.29, 133.16, 132.98, 132.76, 132.73, 132.55, 129.52, 129.36, 126.64, 126.63, 126.42, 117.52, 117.39, 115.38, 115.32, 115.26, 115.15, 115.11, 115.02, 114.99, 112.78, 112.66, 111.20, 111.14, 111.08, 110.99, 110.93, 110.87, 108.01, 107.18, 85.34, 85.30, 67.88, 67.61, 49.49, 49.35, 43.28, 43.00, 38.87, 38.61, 29.44, 29.40, 27.59, 24.98, 24.94, 23.10, 22.69, 22.55, 22.47, 22.37, 22.01, 21.80, 21.53.

Step 7: Synthesis of 1-(3-(6-bromo-7-(difluoromethyl)-3,4-dihydroquinolin-1(2H)-yl)-1-(tetrahydro-2H-pyran-2-yl)-1,4,6,7-tetrahydro-5H-pyrazolo[4,3-c]pyridin-5-yl)ethan-1-one and 1-(3-(6-bromo-7-(difluoromethyl)-3,4-dihydroquinolin-1(2H)-yl)-2-(tetrahydro-2H-pyran-2-yl)-2,4,6,7-tetrahydro-5H-pyrazolo[4,3-c]pyridin-5-yl)ethan-1-one (**11**)

A solution of compound **10** (1.7 g, 1.0 eq) in DCM (30 mL) was treated with NBS (667 mg, 0.95 eq) in portions under ice bath conditions. After 2 h, the reaction mixture was diluted with DCM, washed sequentially with aqueous $Na_2S_2O_3$ solution followed by brine, dried over anhydrous Na_2SO_4, and concentrated under reduced pressure to remove the solvent. The crude product obtained was purified by flash column chromatography (using a gradient of 0-5%

MeOH/DCM) to yield 1-(3-(6-bromo-7-(difluoromethyl)-3,4-dihydroquinolin-1(2H)-yl)-1-(tetrahydro-2H-pyran-2-yl)-1,4,6,7-tetrahydro-5H-pyrazolo[4,3-c]pyridin-5-yl)ethan-1-one and 1-(3-(6-bromo-7-(difluoromethyl)-3,4-dihydroquinolin-1(2H)-yl)-2-(tetrahydro-2H-pyran-2-yl)-2,4,6,7-tetrahydro-5H-pyrazolo[4,3-c]pyridin-5-yl)ethan-1-one (**11**, 1.75 g) as a white foam in a yield of 87%. LC-MS *m/z* $[M+H]^+$ 509.16; ^1H NMR (400 MHz, Chloroform-*d*) δ 7.20 (d, J = 15.9 Hz, 1H), 6.94 – 6.57 (m, 2H), 5.27 – 5.17 (m, 1H), 4.27 – 4.13 (m, 1H), 4.10 – 3.98 (m, 2H), 3.95 – 3.80 (m, 1H), 3.78 – 3.59 (m, 4H), 2.95 – 2.77 (m, 4H), 2.40 – 2.28 (m, 1H), 2.13 – 1.90 (m, 6H), 1.72 – 1.55 (m, 5H).

Steps 8-9: Synthesis of 1-(3-(7-(difluoromethyl)-6-(1-methyl-1H-pyrazol-4-yl)-3,4-dihydroquinolin-1(2H)-yl)-1,4,6,7-tetrahydro-5H-pyrazolo[4,3-c]pyridin-5-yl)ethan-1-one (**13**)

In a mixture comprising intermediate **11** (1.28 g, 1.0 eq), 1-methyl-4-(4,4,5,5-tetramethyl-1,3,2-dioxaborolan-2-yl)-1H-pyrazole (1.04 g, 2.0 eq), $PdCl_2$(dppf) (367 mg, 0.2 eq), and K_2CO_3 (1.38 g, 4.0 eq) in 1,4-dioxane/H_2O (30 mL/5 mL), the components were degassed and purged with nitrogen three times. Subsequently, the mixture was heated at 100 °C for 12 h. LC-MS analysis confirmed the complete consumption of intermediate **11**, with the formation of a primary peak displaying the desired mass spectrum. Upon cooling, the reaction mixture was quenched with water and subjected to extraction with EtOAc. The collected organic layers were washed with brine, dried over anhydrous Na_2SO_4, and concentrated under reduced pressure. The resulting residue underwent purification through flash column chromatography (using a gradient of 0-5% methanol/dichloromethane) to yield a crude product comprising 1-(3-(7-(difluoromethyl)-6-(1-methyl-1H-pyrazol-4-yl)-3,4-dihydroquinolin-1(2H)-yl)-1-(tetrahydro-2H-pyran-2-yl)-1,4,6,7-tetrahydro-5H-pyrazolo[4,3-c]pyridin-5-yl)ethan-1-one and 1-(3-(7-(difluoromethyl)-6-(1-

methyl-1H-pyrazol-4-yl)-3,4-dihydroquinolin-1(2H)-yl)-1-(tetrahydro-2H-pyran-2-yl)-1,4,6,7-tetrahydro-5H-pyrazolo[4,3-c]pyridin-5-yl)ethan-1-one (**12**) as a brownish oil.

The crude intermediate **12** was dissolved in a solution of 3 M HCl in MeOH and stirred at room temperature for 12 h. Subsequently, the reaction mixture was concentrated under reduced pressure, dissolved in DCM, and neutralized with aqueous NaOH to achieve a pH of 7~8. The resulting mixture was extracted with DCM, washed with brine, dried over anhydrous Na_2SO_4, and concentrated under reduced pressure to yield a crude product. This crude product underwent further purification by flash column chromatography (using a gradient of 0-5% MeOH/DCM) to afford 1-(3-(7-(difluoromethyl)-6-(1-methyl-1H-pyrazol-4-yl)-3,4-dihydroquinolin-1(2H)-yl)-1,4,6,7-tetrahydro-5H-pyrazolo[4,3-c]pyridin-5-yl)ethan-1-one (**13**) as a light yellow solid (640 mg, 60% yield from **11**). LC-MS *m/z* $[M+H]^+$ 427.43; ^1H NMR (400 MHz, Methanol-*d*4) δ 7.67 – 7.61 (m, 1H), 7.51 (s, 1H), 7.15 – 7.06 (m, 1H), 6.76 – 6.40 (m, 2H), 4.30 – 4.17 (m, 2H), 3.96 – 3.90 (m, 3H), 3.89 – 3.76 (m, 2H), 3.72 – 3.62 (m, 2H), 2.94 – 2.74 (m, 4H), 2.21 – 2.00 (m, 5H); ^{13}C NMR (101 MHz, MeOD) δ 172.48, 172.38, 148.28, 148.17, 143.92, 143.62, 141.83, 141.47, 139.27, 132.39, 132.27, 131.52, 131.32, 131.11, 130.90, 130.00, 129.75, 124.70, 124.37, 120.54, 118.31, 117.26, 117.20, 115.49, 114.86, 113.67, 112.96, 112.57, 112.52, 106.09, 50.89, 50.79, 44.19, 43.76, 39.84, 39.54, 38.97, 27.95, 27.91, 23.54, 23.45, 22.67, 21.64, 21.35.

Step 10: Synthesis of *trans*-4-(5-acetyl-3-(7-(difluoromethyl)-6-(1-methyl-1H-pyrazol-4-yl)-3,4-dihydroquinolin-1(2H)-yl)-4,5,6,7-tetrahydro-1H-pyrazolo[4,3-c]pyridin-1-yl)cyclohexane-1-carboxylate (**14**)

To a solution of compound **13** (300 mg, 1.0 eq) and compound **2** (658 mg, 3.0 eq) in DMF (5 mL), Cs_2CO_3 (917 mg, 4.0 eq) was added. The resulting mixture was stirred at 70 °C for 7 h. Following the reaction, the mixture was directly subjected to purification by pre-HPLC (45-100%

MeCN (0.1% TFA)/H_2O (0.1% TFA) in 55 min). The desired product began eluting when the MeCN/H_2O ratio reached 52%. The compound *trans*-4-(5-acetyl-3-(7-(difluoromethyl)-6-(1-methyl-1H-pyrazol-4-yl)-3,4-dihydroquinolin-1(2H)-yl)-4,5,6,7-tetrahydro-1H-pyrazolo[4,3-c]pyridin-1-yl)cyclohexane-1-carboxylate (**14**, 257 mg) was isolated as a white solid, yielding 64%. LC-MS: *m/z* $[M+H]^+$ = 567.12; ^1H NMR (400 MHz, $CDCl_3$-*d*) δ 7.64 (s, 1H), 7.46 (s, 1H), 7.08 – 6.97 (m, 1H), 6.83 (s, 1H), 6.47 (td, J = 55.5, 11.6 Hz, 1H), 4.25 (s, 1H), 4.13 (s, 1H), 4.05 – 3.99 (m, 3H), 3.97 – 3.89 (m, 2H), 3.82 – 3.75 (m, 1H), 3.74 – 3.66 (m, 5H), 2.92 – 2.81 (m, 3H), 2.80 – 2.73 (m, 1H), 2.47 – 2.35 (m, 1H), 2.24 – 2.01 (m, 11H), 1.69 – 1.52 (m, 2H); ^{13}C NMR (101 MHz, $CDCl_3$) δ 175.75, 175.71, 171.80, 171.22, 148.60, 148.01, 142.27, 142.20, 137.79, 137.46, 137.30, 136.57, 131.35, 130.72, 130.50, 130.00, 129.94, 129.79, 129.73, 129.58, 129.52, 126.68, 126.60, 120.19, 120.08, 119.87, 115.93, 115.84, 113.57, 113.49, 111.18, 110.65, 105.94, 105.39, 57.65, 57.56, 51.78, 49.91, 49.78, 43.47, 43.17, 41.79, 41.73, 39.42, 39.31, 38.56, 38.49, 31.32, 28.10, 28.07, 27.38, 27.34, 22.71, 22.17, 22.08, 21.82, 21.09, 20.75.

Step 11: Synthesis of *trans*-4-(5-acetyl-3-(7-(difluoromethyl)-6-(1-methyl-1H-pyrazol-4-yl)-3,4-dihydroquinolin-1(2H)-yl)-4,5,6,7-tetrahydro-1H-pyrazolo[4,3-c] 21 yridine-1-yl)cyclohexane-1-carbaldehyde (**15**)

Compound **14** (232 mg, 1.0 eq) was dissolved in anhydrous DCM (15 mL), and the solution was degassed by purging with nitrogen three times. DIBAL (25% in toluene, 1.1 mL, 4.0 eq) was then added dropwise at -78 °C over 1 h. The reaction mixture was stirred at -78 °C for an additional 2 h. Subsequently, the reaction was quenched with aqueous potassium sodium tartrate and warmed to room temperature. The resulting mixture was extracted with DCM, and the organic layers were washed with brine, dried over anhydrous Na_2SO_4, filtered, and concentrated under reduced pressure. The crude residue was purified by pre-HPLC (35-100% MeCN/H_2O in 65 min). The

desired product began eluting when the MeCN/H_2O ratio reached 44%. The compound *trans*-4-(5-acetyl-3-(7-(difluoromethyl)-6-(1-methyl-1H-pyrazol-4-yl)-3,4-dihydroquinolin-1(2H)-yl)-4,5,6,7-tetrahydro-1H-pyrazolo[4,3-c]pyridin-1-yl)cyclohexane-1-carbaldehyde (**15**) was isolated as a white solid (85 mg, 39% yield). LC-MS: *m/z* $[M+H]^+$ 537.10; ^1H NMR (400 MHz, $CDCl_3$-*d*) δ 9.69 (d, J = 3.5 Hz, 1H), 7.56 – 7.51 (m, 1H), 7.43 – 7.37 (m, 1H), 7.07 – 6.96 (m, 1H), 6.89 – 6.83 (m, 1H), 6.51 (td, J = 55.6, 10.9 Hz, 1H), 4.25 (s, 1H), 4.12 (s, 1H), 3.98 – 3.93 (m, 3H), 3.93 – 3.82 (m, 2H), 3.78 – 3.64 (m, 3H), 2.91 – 2.82 (m, 2H), 2.81 – 2.69 (m, 2H), 2.40 – 2.28 (m, 1H), 2.24 – 2.00 (m, 11H), 1.51 – 1.36 (m, 2H); ^{13}C NMR (101 MHz, $CDCl_3$) δ 203.48, 171.88, 171.38, 148.46, 147.96, 142.12, 142.00, 138.07, 137.11, 137.07, 136.93, 131.39, 130.97, 130.73, 129.97, 129.76, 129.52, 126.87, 126.75, 120.09, 119.89, 115.88, 115.80, 113.53, 113.45, 111.39, 111.10, 110.77, 108.09, 105.82, 105.33, 57.74, 57.62, 53.97, 49.89, 49.78, 48.58, 48.52, 43.37, 43.07, 39.35, 39.21, 38.43, 38.38, 30.94, 27.25, 27.20, 25.01, 22.59, 22.08, 22.01, 21.73, 20.96, 20.60.

Step 12: Synthesis of 5-(4-(((1r,4r)-4-(5-acetyl-3-(7-(difluoromethyl)-6-(1-methyl-1H-pyrazol-4-yl)-3,4-dihydroquinolin-1(2H)-yl)-4,5,6,7-tetrahydro-1H-pyrazolo[4,3-c]pyridin-1-yl)cyclohexyl)methyl)piperazin-1-yl)-2-(2,6-dioxopiperidin-3-yl)isoindoline-1,3-dione (**16**, **CBPD-409**)

NaBH(OAc)$_3$ (117 mg, 3.0 eq) was added in portions over 2 h to a mixture of **15** (100 mg, 1.0 eq) and **4** (110 mg, 1.3 eq) suspended in DCE/DMF (8 mL/4 mL). The reaction was then stirred at room temperature for 12 h. After completion, the mixture was concentrated under reduced pressure. The resulting residue was subjected to purification by pre-HPLC (30-100% MeCN (0.1% TFA) /H_2O (0.1% TFA) in 70 min). The final compound, 5-(4-(((1r,4r)-4-(5-acetyl-3-(7-(difluoromethyl)-6-(1-methyl-1H-pyrazol-4-yl)-3,4-dihydroquinolin-1(2H)-yl)-4,5,6,7-

tetrahydro-1H-pyrazolo[4,3-c]pyridin-1-yl)cyclohexyl)methyl)piperazin-1-yl)-2-(2,6-dioxopiperidin-3-yl)isoindoline-1,3-dione (**16**, **CBPD-409**), was obtained as a light yellow solid (78 mg, 48% yield). UPLC-MS: 1.69 min, purity > 95%; MS (ESI) *m/z* calcd. For $C_{46}H_{52}F_2N_{10}O_5$ $[M + H]^+$ 863.42, found 863.43; HRMS (APCI) *m/z* calcd. For $C_{46}H_{52}F_2N_{10}O_5$ $[M + H]^+$ 863.4163, found 863.4189; ^1H NMR (400 MHz, MeOH-d_4) δ 7.77 (d, J = 8.5 Hz, 1H), 7.64 (s, 1H), 7.54 – 7.46 (m, 2H), 7.36 (dd, J = 8.5, 2.3 Hz, 1H), 7.11 (d, J = 8.9 Hz, 1H), 6.77 – 6.40 (m, 2H), 5.10 (dd, J = 12.5, 5.4 Hz, 1H), 4.31 – 4.03 (m, 5H), 3.97 – 3.78 (m, 6H), 3.67 (q, J = 6.7 Hz, 4H), 3.51 – 3.34 (m, 3H), 3.18 (d, J = 6.3 Hz, 2H), 2.97 – 2.79 (m, 5H), 2.79 – 2.65 (m, 2H), 2.19 (s, 2H), 2.16 – 1.97 (m, 11H), 1.45 – 1.27 (m, 2H); ^{13}C NMR (101 MHz, MeOD) δ 174.57, 172.40, 172.26, 171.62, 168.96, 168.67, 155.71, 150.22, 149.77, 143.82, 139.82, 139.33, 139.07, 135.49, 132.28, 132.16, 131.25, 131.23, 131.00, 130.79, 127.51, 127.37, 126.12, 122.76, 122.22, 121.22, 120.89, 120.79, 120.47, 118.36, 117.39, 115.50, 115.05, 112.71, 112.64, 111.47, 111.03, 110.97, 110.41, 107.57, 63.43, 58.16, 52.98, 51.11, 51.03, 50.54, 45.86, 44.60, 43.89, 40.16, 39.66, 38.93, 32.95, 32.47, 32.19, 30.50, 28.44, 23.71, 23.38, 23.25, 22.59, 21.67, 21.38.

3. Method and procedure for the preparation of CBPD-409-Me

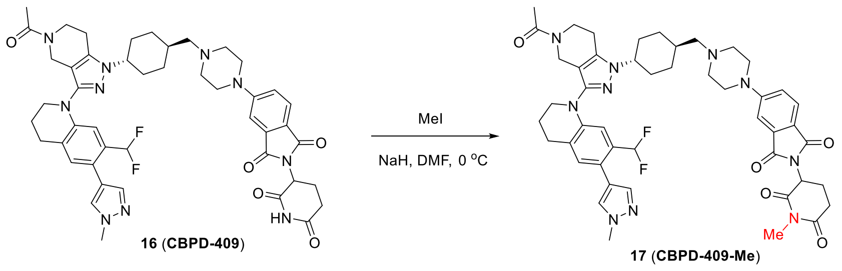

To a solution of **16** (10.8 mg, 1.0 eq) in DMF (1 mL) was added NaH (4.4 mg, 10.0 eq) under ice bath condition, followed by MeI (3.44 µL, 5.0 eq). The reaction was stirred for 30 min, then quenched with TFA and water. The resulting mixture was directly purified by pre-HPLC (30-100% MeCN/H_2O in 70 min) to give product 5-(4-(((1r,4r)-4-(5-acetyl-3-(7-(difluoromethyl)-6-

(1-methyl-1H-pyrazol-4-yl)-3,4-dihydroquinolin-1(2H)-yl)-4,5,6,7-tetrahydro-1H-pyrazolo[4,3-c]pyridin-1-yl)cyclohexyl)methyl)piperazin-1-yl)-2-(1-methyl-2,6-dioxopiperidin-3-yl)isoindoline-1,3-dione (**17**, **CBPD-409-Me**) as a light yellow solid (9.0 mg, 82% yield): UPLC-MS: 1.47 min, purity > 95%; MS (ESI) *m/z* calcd. For $C_{47}H_{54}F_2N_{10}O_5$ $[M + H]^+$ 877.43, found 866.80; ^{1}H NMR (400 MHz, Methanol-d_4) δ 7.77 (d, J = 8.5 Hz, 1H), 7.64 (s, 1H), 7.54 – 7.47 (m, 2H), 7.37 (dd, J = 8.5, 2.3 Hz, 1H), 7.15 – 7.07 (m, 1H), 6.79 – 6.40 (m, 2H), 5.12 (dd, J = 12.9, 5.4 Hz, 1H), 4.32 – 4.02 (m, 5H), 3.97 – 3.79 (m, 6H), 3.78 – 3.59 (m, 4H), 3.56 – 3.32 (m, 3H), 3.22 – 3.11 (m, 5H), 2.98 – 2.79 (m, 6H), 2.76 – 2.63 (m, 1H), 2.19 (s, 2H), 2.14 – 1.98 (m, 11H), 1.43 – 1.32 (m, 2H).

4. UPLC-MS spectra

The UPLC-MS spectrum for compound 16 (CBPD-409)

SAMPLE INFORMATION

Sample Name:	ZXC-409-Weeks	Acquired By:	System
Sample Type:	Unknown	Sample Set Name:	1
Vial:	1:D,8	Acq. Method Set:	10to100Bin5min_noDelay
Injection #:	1	Processing Method:	Default
Injection Volume:	6.00 ul	Channel Name:	254.0nm
Run Time:	5.0 Minutes	Proc. Chnl. Descr.:	PDA Spectrum PDA 254.0 nm
Date Acquired:	7/29/2022 10:08:59 PM EDT		
Date Processed:	7/29/2022 10:23:11 PM EDT		

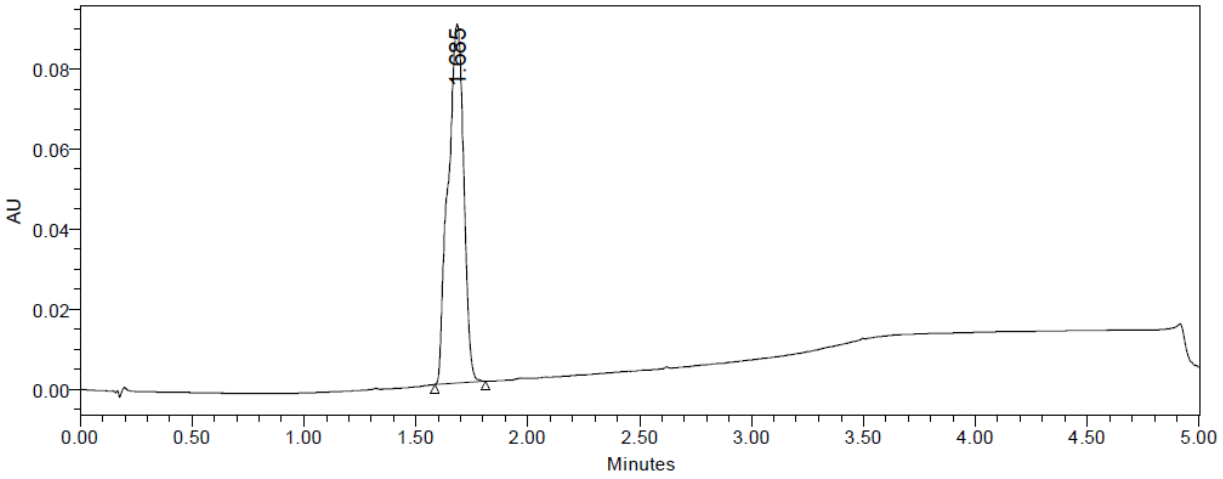

	RT	Area	% Area	Height
1	1.685	433496	100.00	89466

The UPLC-MS spectrum for compound 17 (CBPD-409-Me)

SAMPLE INFORMATION

Sample Name:	ZXC-7-261-P	Acquired By:	System
Sample Type:	Unknown	Date Acquired:	5/7/2023 1:28:57 AM EDT
Vial:	1:A,4	Acq. Method Set:	New10to 100% B 5 min_NoDelay
Injection #:	1	Date Processed:	5/7/2023 3:14:43 AM EDT
Injection Volume:	6.00 ul	Processing Method:	Bruce1
Run Time:	5.0 Minutes	Channel Name:	254.0nm
Sample Set Name:	3	Proc. Chnl. Descr.:	PDA Spectrum PDA 254.0 nm (PDA

Auto-Scaled Chromatogram

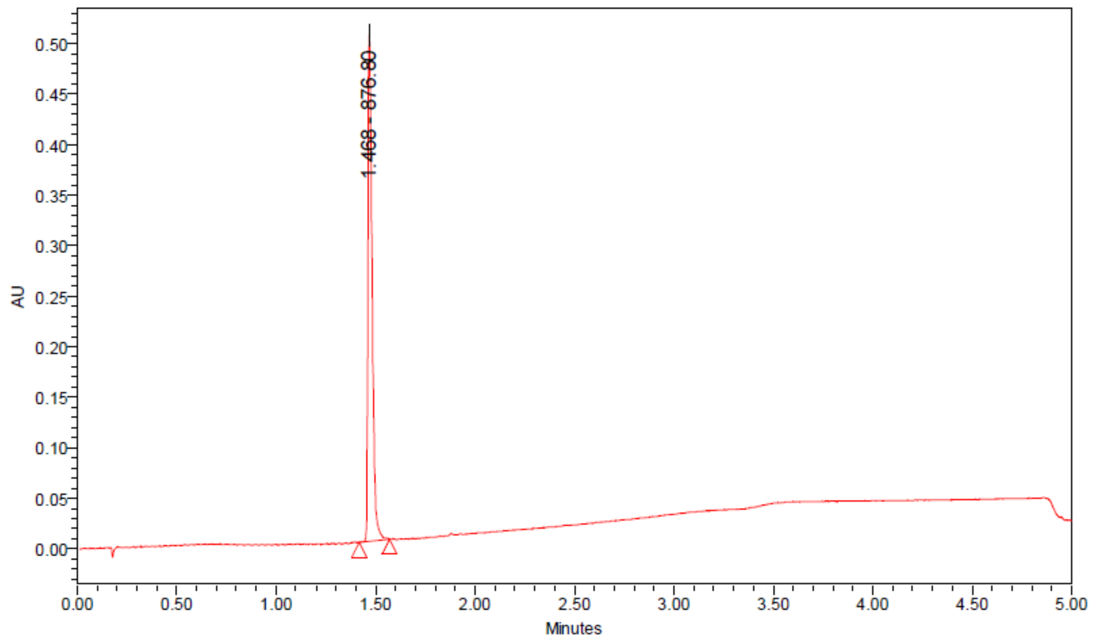

Peak Results

	RT	Area	Height	% Area
1	1.468	741778	501466	100.00

Peak Results

	Base Peak (m/z)
1	876.80

5. NMR spectra

^{1}H-NMR spectrum for compound 2

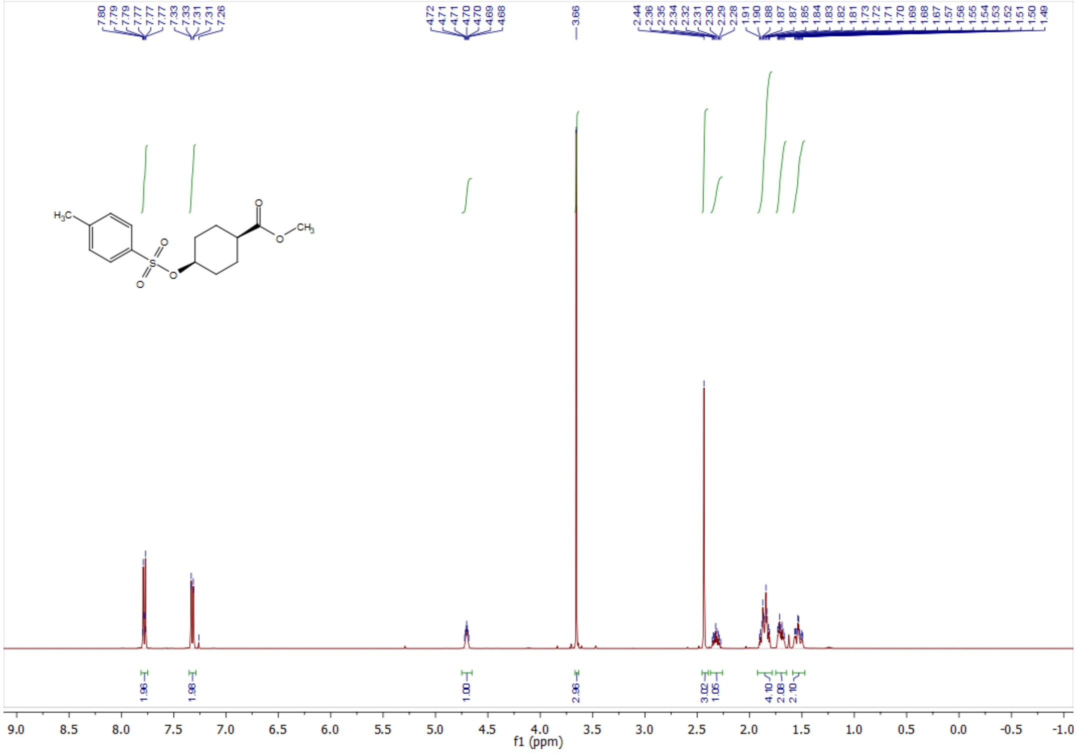

^{1}H-NMR spectrum for compound 4

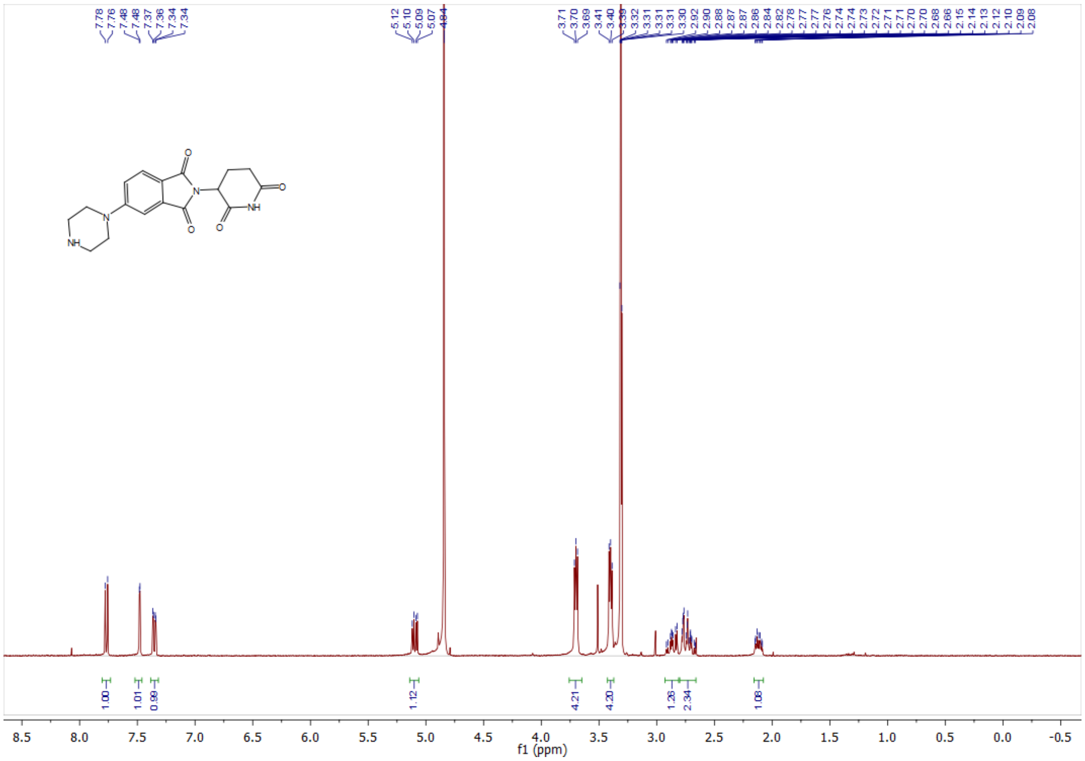

^{1}H-NMR spectrum for compound 7

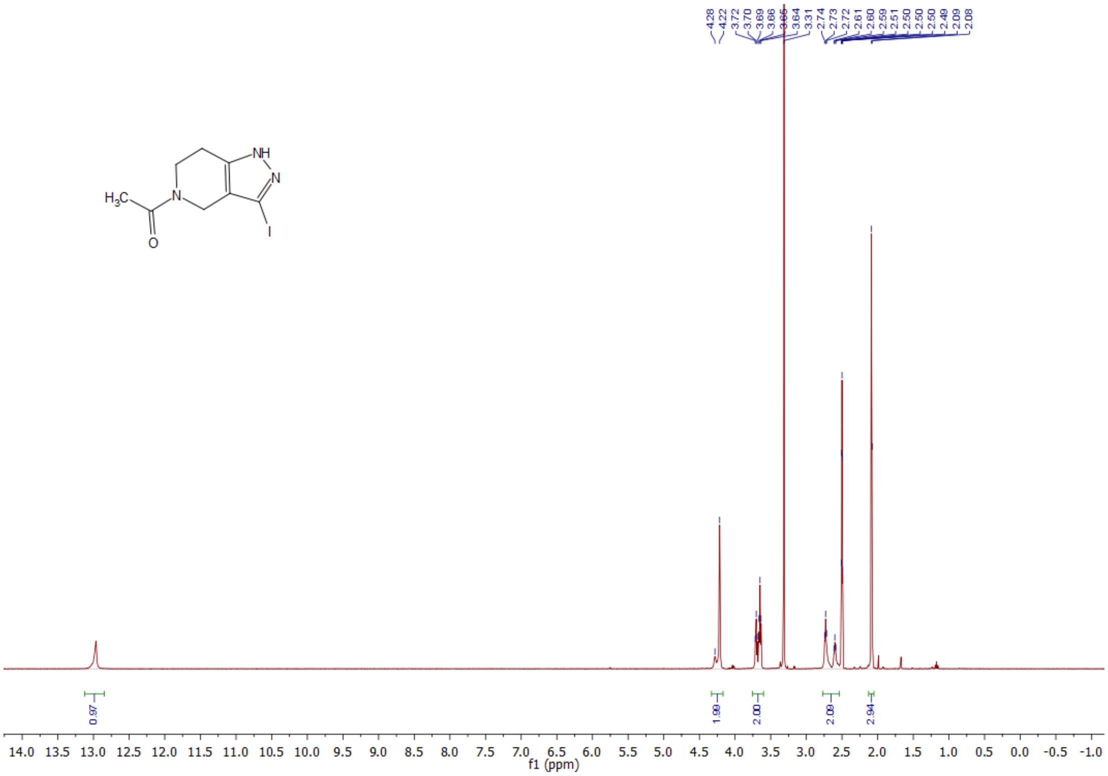

^{1}H-NMR spectrum for compound 8

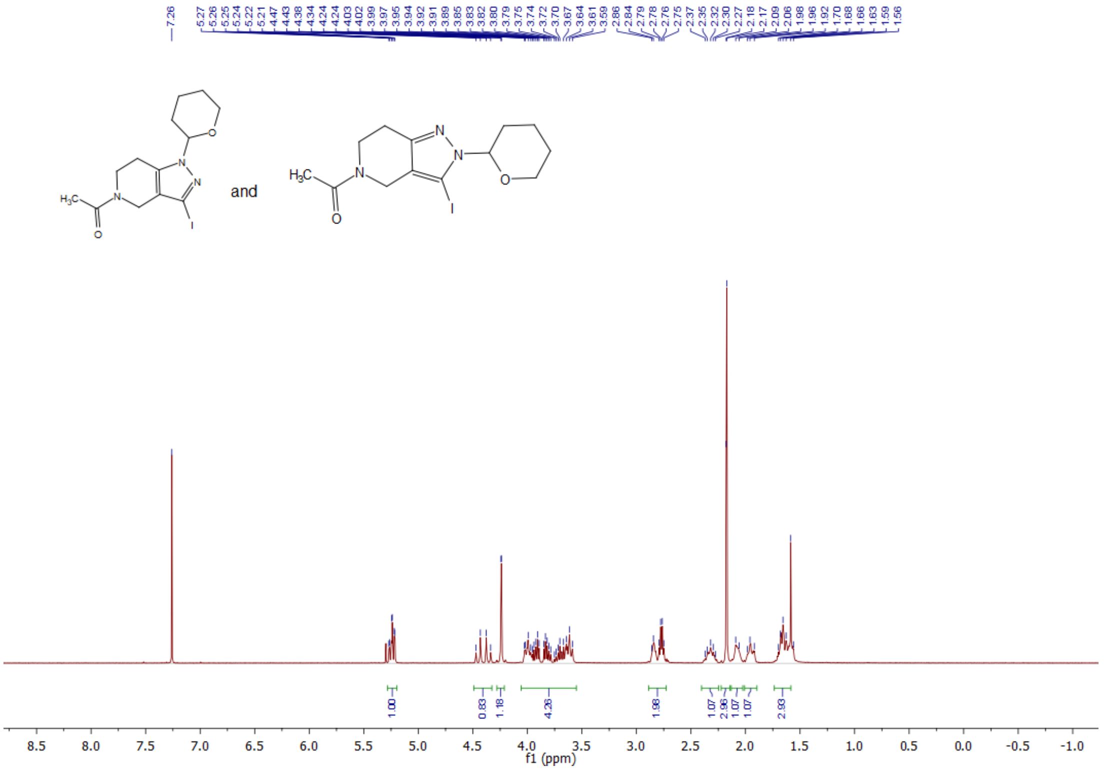

^{1}H-NMR spectrum for compound 10

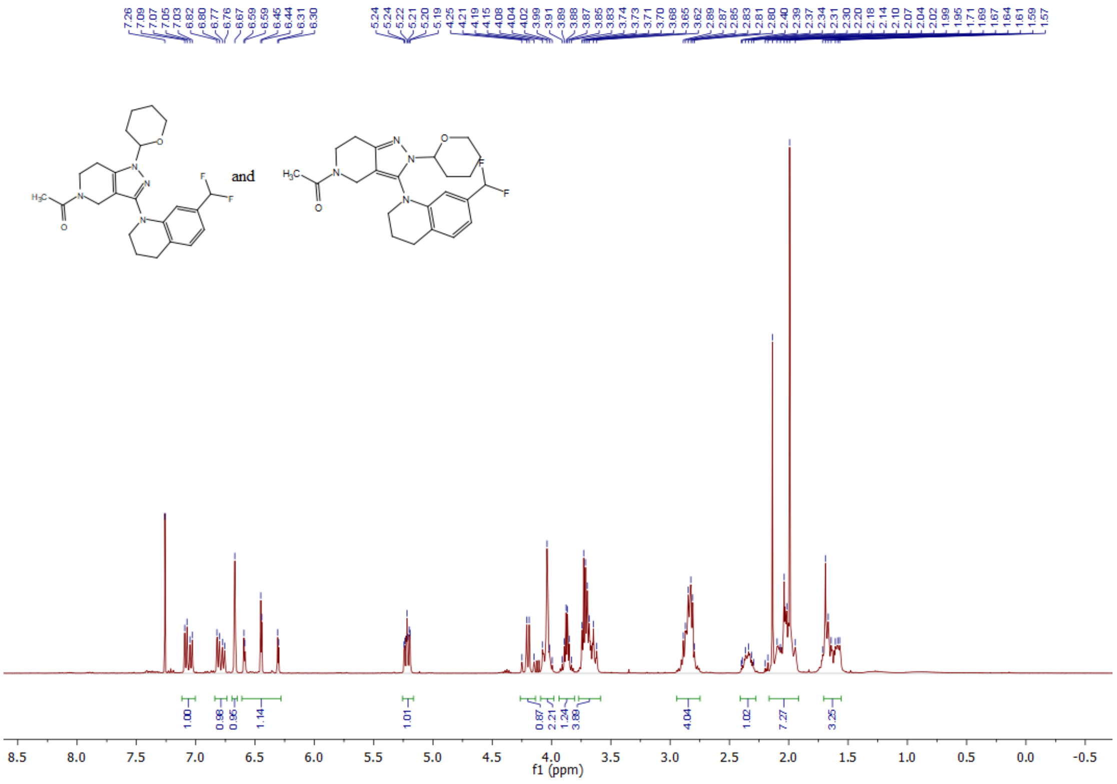

^{13}C-NMR spectrum for compound 10

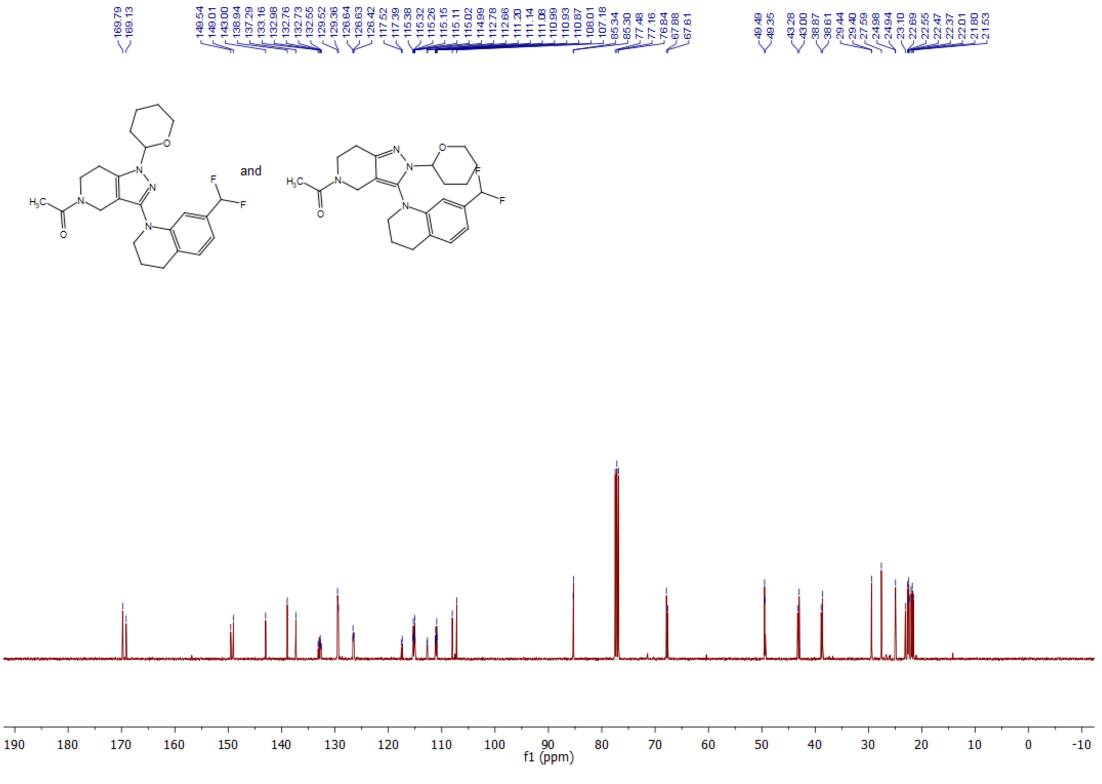

^{1}H-NMR spectrum for compound 13

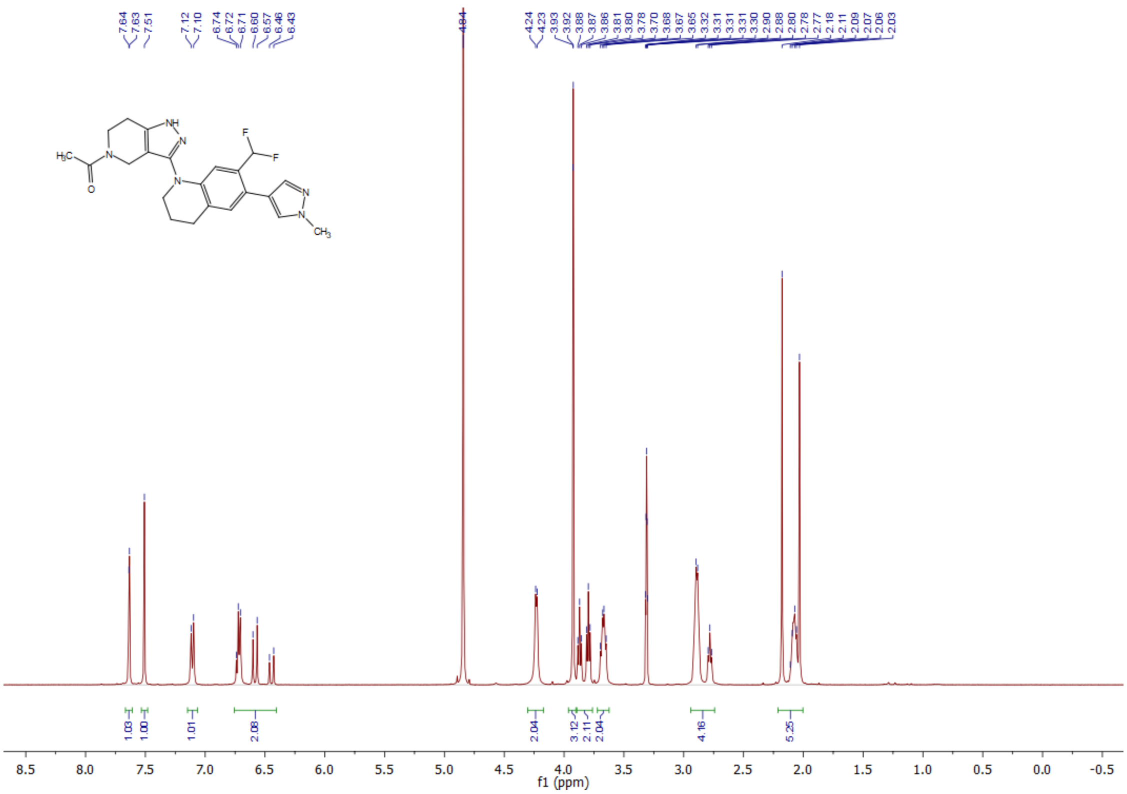

^{13}C-NMR spectrum for compound 13

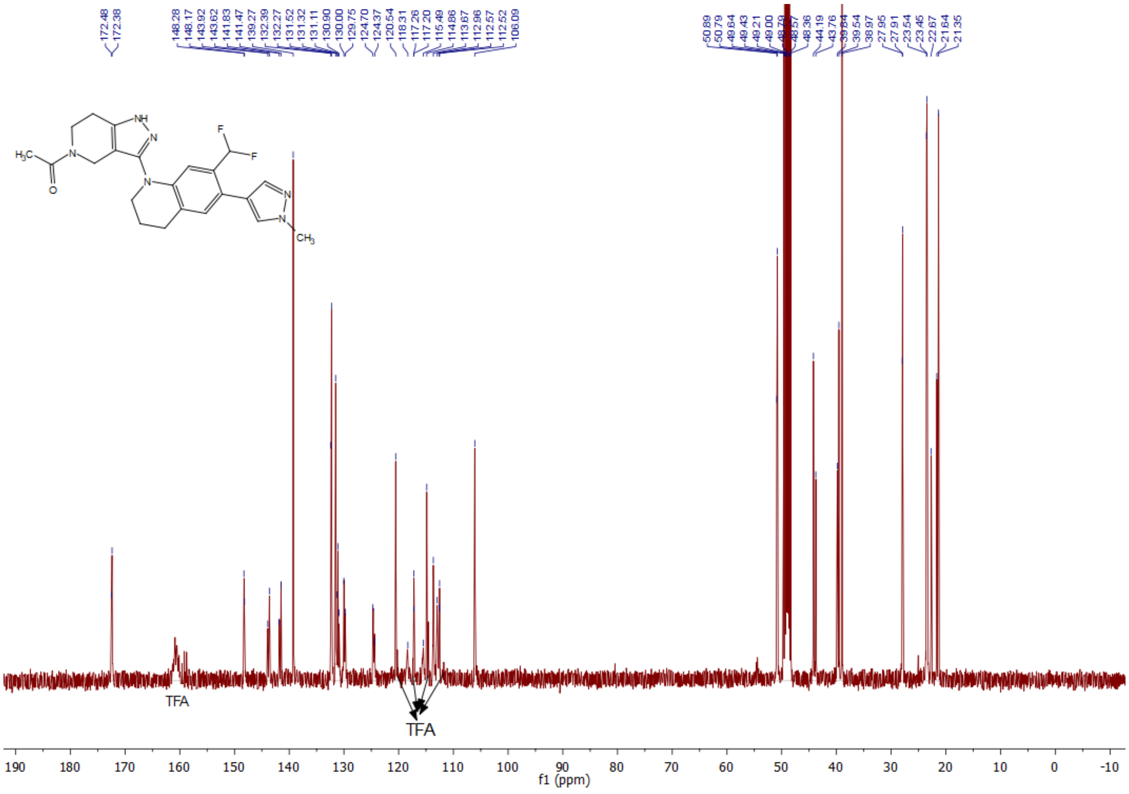

^{1}H-NMR spectrum for compound 14

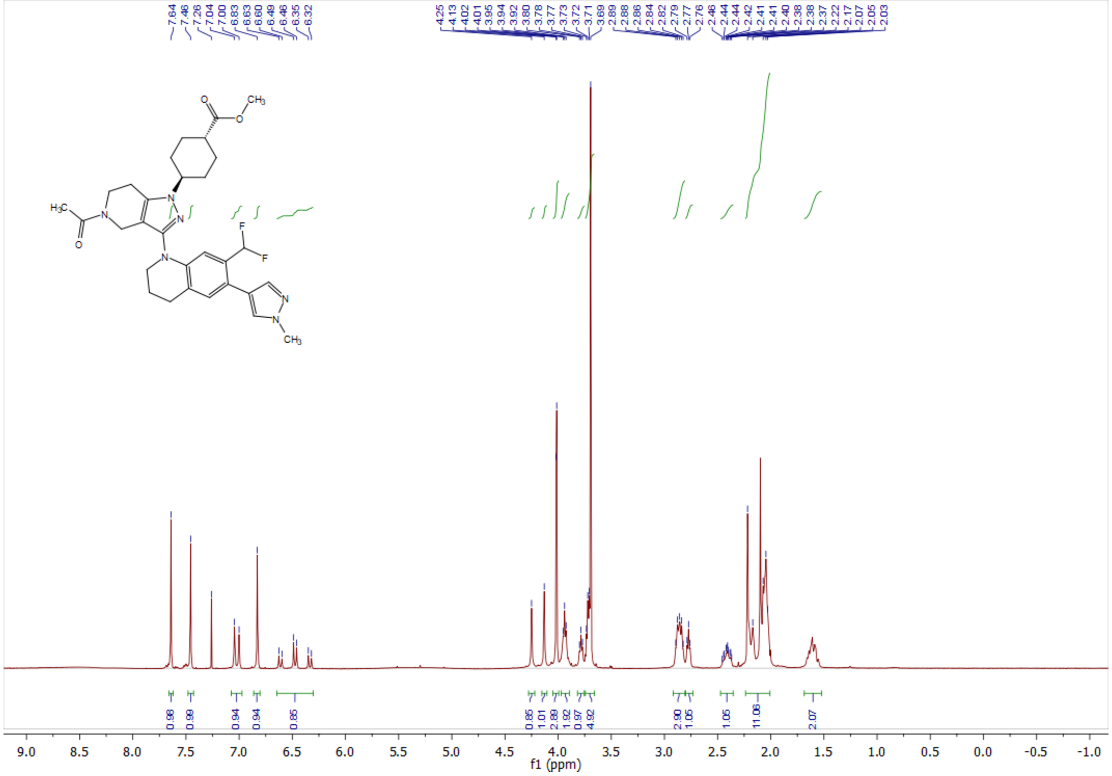

^{13}C-NMR spectrum for compound 14

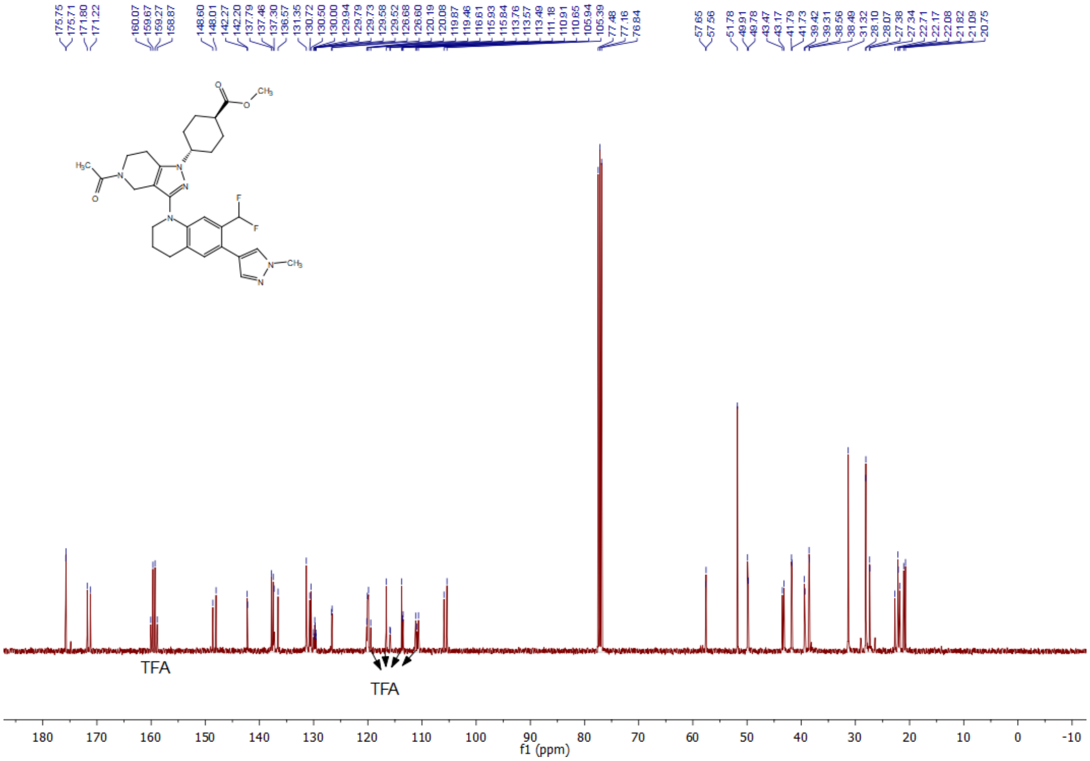

^{1}H-NMR spectrum for compound 15

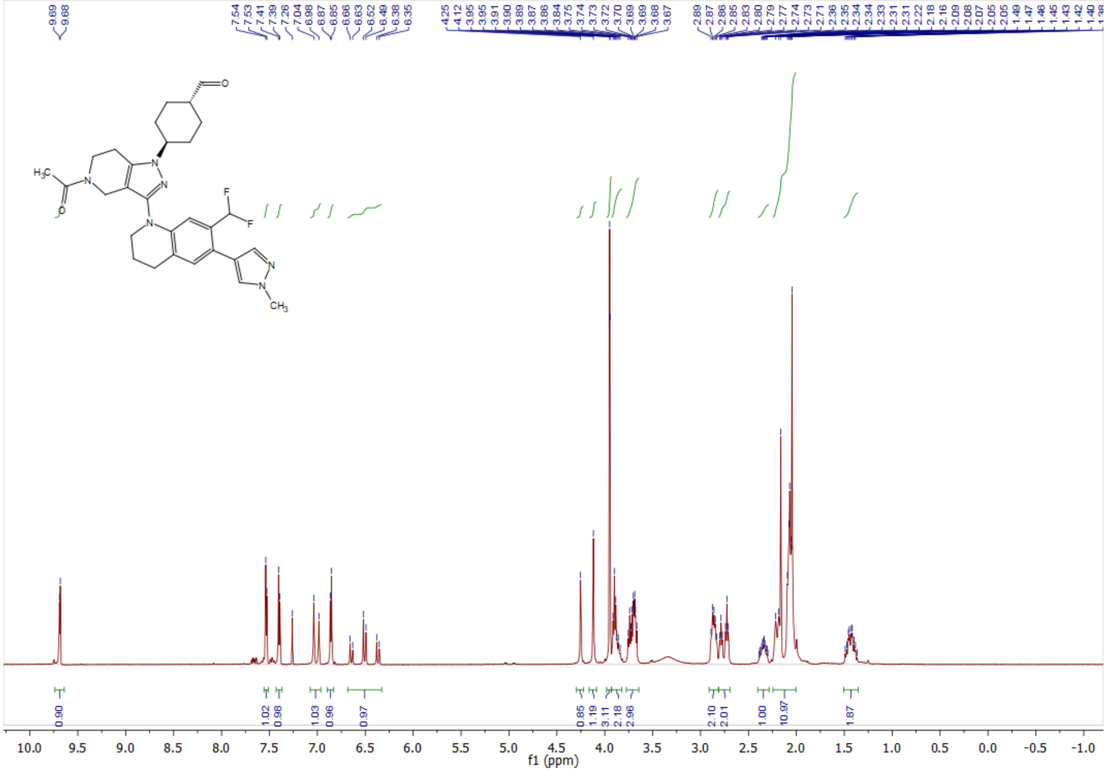

^{1}H-NMR spectrum for compound 16 (CBPD-409)

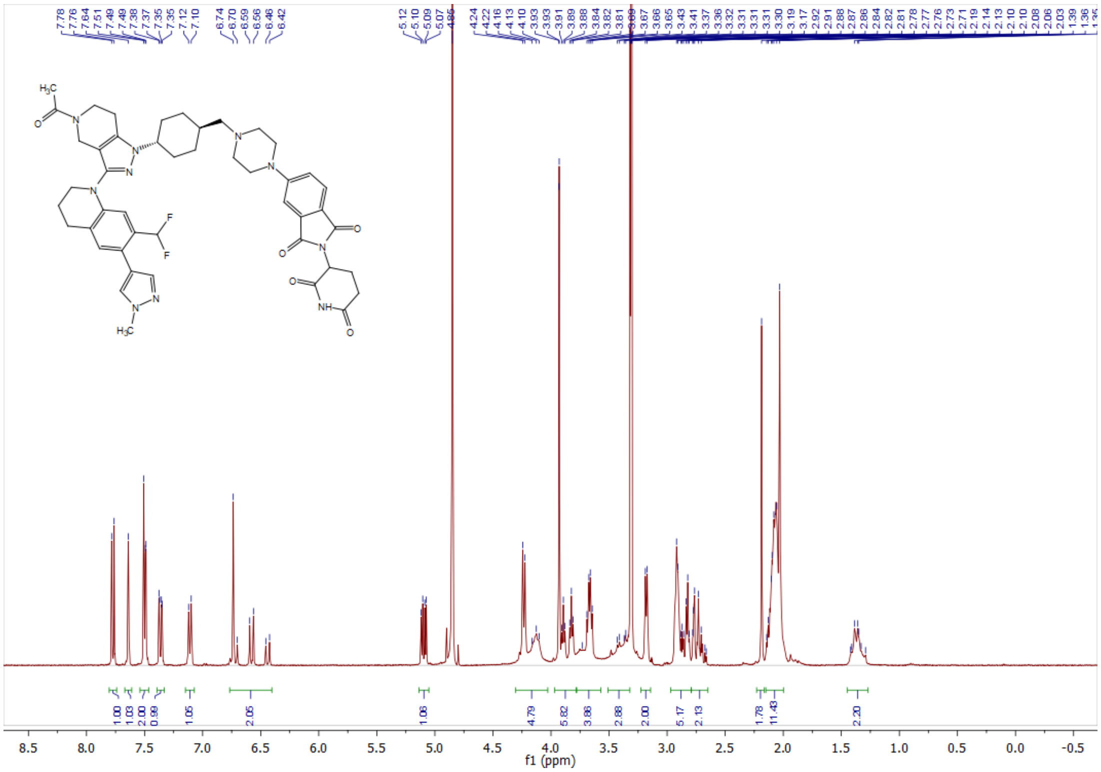

^{13}C-NMR spectrum for compound 16 (CBPD-409)

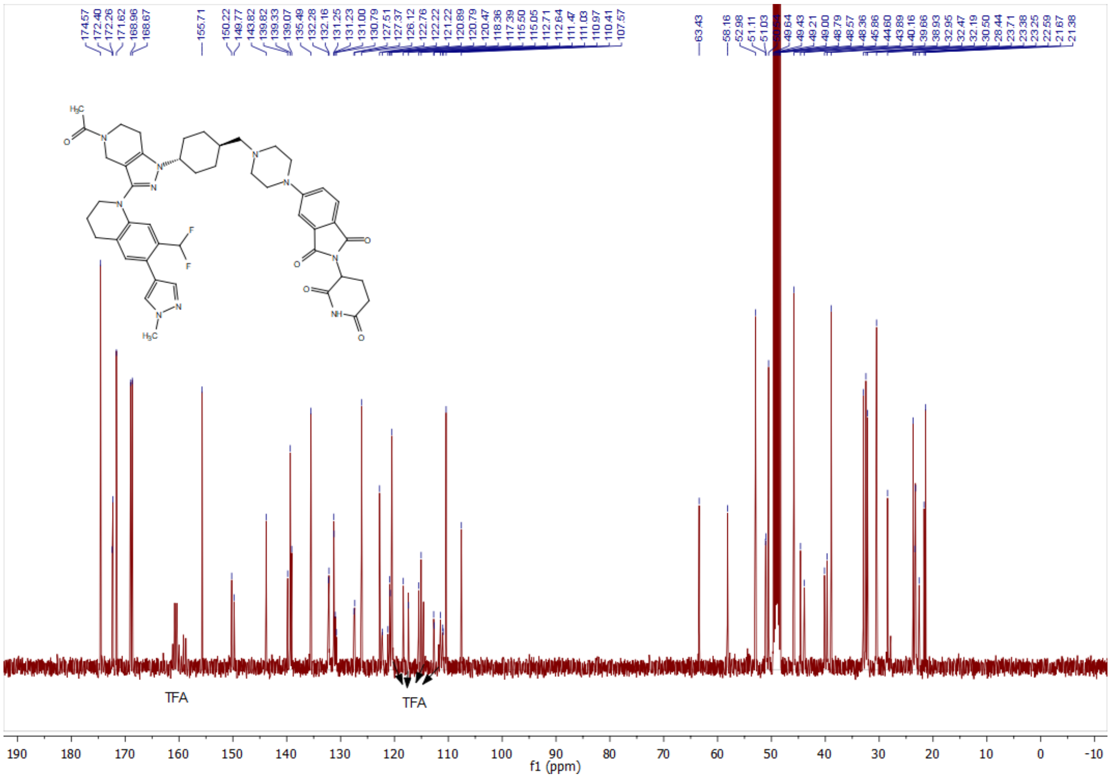

^{1}H-NMR spectrum for compound 17 (CBPD-409-Me)

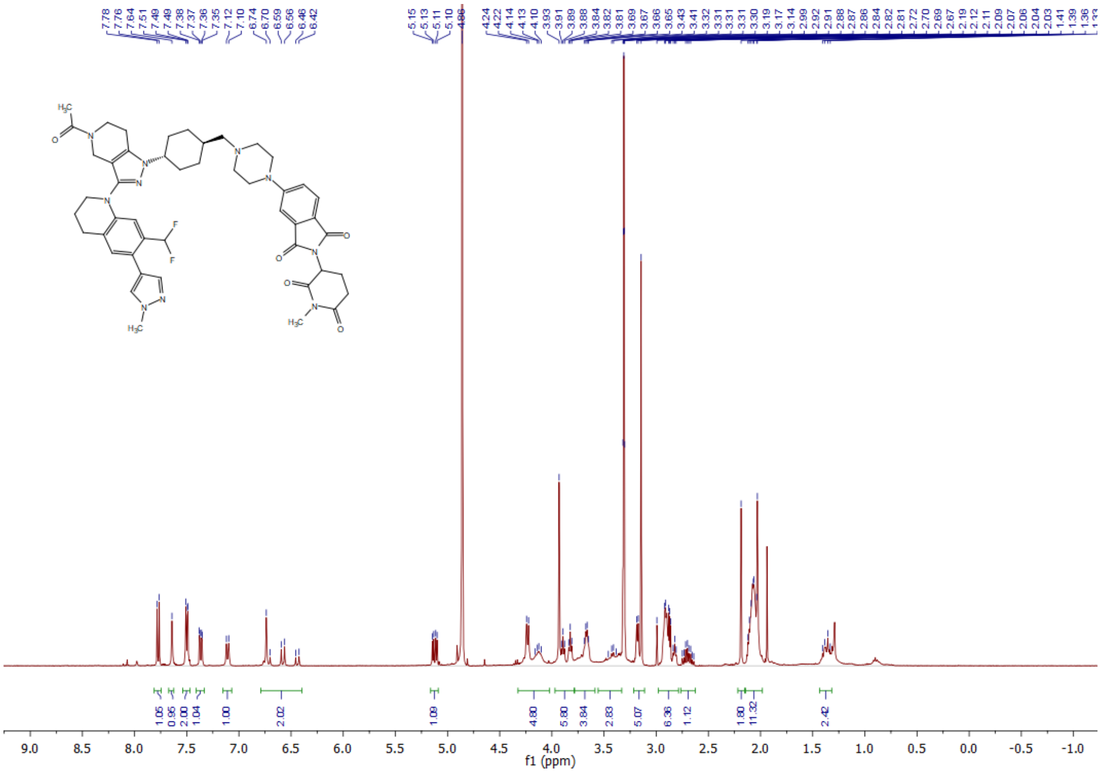

References

1. Xiao, L. *et al.* Targeting SWI/SNF ATPases in enhancer-addicted prostate cancer. *Nature* **601**, 434-439 (2022).
2. Harrow, J. *et al.* GENCODE: the reference human genome annotation for The ENCODE Project. *Genome Res* **22**, 1760-74 (2012).
3. Li, H. & Durbin, R. Fast and accurate short read alignment with Burrows-Wheeler transform. *Bioinformatics* **25**, 1754-60 (2009).
4. Danecek, P. *et al.* Twelve years of SAMtools and BCFtools. *Gigascience* **10**(2021).
5. Ramirez, F. *et al.* deepTools2: a next generation web server for deep-sequencing data analysis. *Nucleic Acids Res* **44**, W160-5 (2016).
6. Liao, Y., Smyth, G.K. & Shi, W. featureCounts: an efficient general purpose program for assigning sequence reads to genomic features. *Bioinformatics* **30**, 923-30 (2014).
7. Kent, W.J., Zweig, A.S., Barber, G., Hinrichs, A.S. & Karolchik, D. BigWig and BigBed: enabling browsing of large distributed datasets. *Bioinformatics* **26**, 2204-7 (2010).
8. Yu, G., Wang, L.G. & He, Q.Y. ChIPseeker: an R/Bioconductor package for ChIP peak annotation, comparison and visualization. *Bioinformatics* **31**, 2382-3 (2015).
9. Zhu, L.J. Integrative analysis of ChIP-chip and ChIP-seq dataset. *Methods Mol Biol* **1067**, 105-24 (2013).
10. Zhang, Y. *et al.* Model-based analysis of ChIP-Seq (MACS). *Genome Biol* **9**, R137 (2008).
11. Pomerantz, M.M. *et al.* The androgen receptor cistrome is extensively reprogrammed in human prostate tumorigenesis. *Nat Genet* **47**, 1346-51 (2015).
12. Pomerantz, M.M. *et al.* Prostate cancer reactivates developmental epigenomic programs during metastatic progression. *Nat Genet* **52**, 790-799 (2020).
13. Lawrence, M. *et al.* Software for computing and annotating genomic ranges. *PLoS Comput Biol* **9**, e1003118 (2013).
14. Palanisamy, N. *et al.* The MD Anderson Prostate Cancer Patient-derived Xenograft Series (MDA PCa PDX) Captures the Molecular Landscape of Prostate Cancer and Facilitates Marker-driven Therapy Development. *Clin Cancer Res* **26**, 4933-4946 (2020).
15. Jafari, O., Babaei, H., Kheirandish, R., Samimi, A.S. & Zahmatkesh, A. Histomorphometric evaluation of mice testicular tissue following short- and long-term effects of lipopolysaccharide-induced endotoxemia. *Iran J Basic Med Sci* **21**, 47-52 (2018).
16. Johnsen, S.G. Testicular biopsy score count--a method for registration of spermatogenesis in human testes: normal values and results in 335 hypogonadal males. *Hormones* **1**, 2-25 (1970).
