# Supplementary material for: Targeting histone H2B acetylated enhanceosomes via p300/CBP degradation in prostate cancer
Source: Nat Genet. 2025 Oct 3;57(10):2468–81. doi: 10.1038/s41588-025-02336-6 (PMC12513837; doi:10.1038/s41588-025-02336-6)
Supplement: Supplementary file 2 — Reporting Summary [file 41588_2025_2336_MOESM2_ESM.pdf]

nature portfolio

Corresponding author(s): Arul M. Chinnaiyan

Last updated by author(s): Jul 10, 2025

Reporting Summary

Nature Portfolio wishes to improve the reproducibility of the work that we publish. This form provides structure for consistency and transparency in reporting. For further information on Nature Portfolio policies, see our Editorial Policies and the Editorial Policy Checklist.

Statistics

For all statistical analyses, confirm that the following items are present in the figure legend, table legend, main text, or Methods section.

n/a	Confirmed	
☐	☒	The exact sample size (*n*) for each experimental group/condition, given as a discrete number and unit of measurement
☐	☒	A statement on whether measurements were taken from distinct samples or whether the same sample was measured repeatedly
☐	☒	The statistical test(s) used AND whether they are one- or two-sided<br>*Only common tests should be described solely by name; describe more complex techniques in the Methods section.*
☐	☒	A description of all covariates tested
☐	☒	A description of any assumptions or corrections, such as tests of normality and adjustment for multiple comparisons
☐	☒	A full description of the statistical parameters including central tendency (e.g. means) or other basic estimates (e.g. regression coefficient) AND variation (e.g. standard deviation) or associated estimates of uncertainty (e.g. confidence intervals)
☐	☒	For null hypothesis testing, the test statistic (e.g. *F*, *t*, *r*) with confidence intervals, effect sizes, degrees of freedom and *P* value noted<br>*Give P values as exact values whenever suitable.*
☒	☐	For Bayesian analysis, information on the choice of priors and Markov chain Monte Carlo settings
☒	☐	For hierarchical and complex designs, identification of the appropriate level for tests and full reporting of outcomes
☐	☒	Estimates of effect sizes (e.g. Cohen's *d*, Pearson's *r*), indicating how they were calculated

Our web collection on statistics for biologists contains articles on many of the points above.

Software and code

Policy information about availability of computer code

Data collection	No software was used for data collection.
Data analysis	Computational tools used:<br>GraphPad Prism 9 and in-built statistical tools<br>PICARD Mark Duplicates (version 2.9.0)<br>HOMER (version v.4.10)<br>MACS2 (version 2.1.1.20160309)<br>BWA (version 0.7.17-r1198-dirty)<br>EdgeR (version 3.34.1)<br>EnhancedVolcano (version 1.15.0)<br>Limma (version 3.58.1.)<br>Fgsea (version 1.28.0)<br>Gtable (version 0.3.5)<br>Kallisto (version 0.50.1)<br>Gplots (version 3.1.3.1)<br>Trimmomatic (version 0.39)<br>Samtools (version 1.19)<br>Bedtools (version 2.31.1)<br>Deeptools (version 3.5.4)<br>ChIPpeakAnno (version 3.6.5)<br>ChIPseeker (version 1.35.3)

Lranges (version 2.36.0)
Tidyverse (v2.0.0)
Ggplot2 (v3.5.1)

For manuscripts utilizing custom algorithms or software that are central to the research but not yet described in published literature, software must be made available to editors and reviewers. We strongly encourage code deposition in a community repository (e.g. GitHub). See the Nature Portfolio guidelines for submitting code & software for further information.

Data

Policy information about availability of data

All manuscripts must include a data availability statement. This statement should provide the following information, where applicable:

- Accession codes, unique identifiers, or web links for publicly available datasets
- A description of any restrictions on data availability
- For clinical datasets or third party data, please ensure that the statement adheres to our policy

All data are available in the manuscript and the supplementary information. All sequencing data generated in this study have been deposited in the following National Center for Biotechnology Information Gene Expression Omnibus (NCBI GEO) repository: GSE255134. ChIP-seq datasets for normal, primary PCa, and mCRPC were obtained from GEO accessions GSE130408 and GSE70079. Gene dependency scores were analyzed using CRISPR knockout datasets available from the DepMap portal (https://depmap.org). The proteomics data have been deposited to the ProteomeXchange Consortium via the PRIDE partner repository with the dataset identifier PXD065228 for TMT-mass spectrometry and PXD065292 for acetyl-lysine proteomics.

Research involving human participants, their data, or biological material

Policy information about studies with human participants or human data. See also policy information about sex, gender (identity/presentation), and sexual orientation and race, ethnicity and racism.

Reporting on sex and gender	All archival tissues come from male patients that were treated for prostate cancer at the the University of Michigan Hospital in Ann Arbor, Michigan.
Reporting on race, ethnicity, or other socially relevant groupings	All patients are Caucasian white men who received treatment in North America.
Population characteristics	The human samples used in this study were archival, formalin-fixed paraffin-embedded (FFPE) prostate cancer specimens obtained from the departmental archive. All samples were fully de-identified prior to analysis. As prostate cancer occurs in males, the sex of all participants is male by definition. Other covariate information (e.g., age, clinical history) was not available or applicable due to the de-identified nature of the samples.
Recruitment	Patients' tissues were taken from Pathology archives at the University of Michigan Hospital in Ann Arbor, Michigan.
Ethics oversight	The University of Michigan Institutional Review Board approved the acquisition and use of clinical FFPE specimens from the pathology archives in this study.

Note that full information on the approval of the study protocol must also be provided in the manuscript.

Field-specific reporting

Please select the one below that is the best fit for your research. If you are not sure, read the appropriate sections before making your selection.

☒ Life sciences ☐ Behavioural & social sciences ☐ Ecological, evolutionary & environmental sciences

For a reference copy of the document with all sections, see nature.com/documents/nr-reporting-summary-flat.pdf

Life sciences study design

All studies must disclose on these points even when the disclosure is negative.

Sample size	Sample sizes were empirically and statistically determined. For animal experiments, n=10-20 tumors were used for the pilot and efficacy studies. Using >10 tumors per treatment group, the statistical power to detect a 50% decrease in the mean tumor volume or metastatic burden in the treatment group is estimated to be 92.3% if the coefficient of variation (CV) is 40%. All in vitro experiments were performed with at least 3 technical replicates across two independent experiments. All samples sizes for various assays are listed in the Methods section or the figure legends. For in vitro experiments, no formal statistical method was used to predetermine sample size. Instead, sample sizes were selected based on empirical experience and commonly accepted standards in the field to ensure reproducibility and adequate statistical power.<br>All in vitro experiments were performed with at least three technical replicates and repeated across at least two independent experiments. These sample sizes are considered sufficient to detect robust and reproducible biological effects. Sample sizes for each assay, including replicates and experimental design, are detailed in the Methods section and figure legends.

Data exclusions	No data were excluded from the published publicly-available patient sequencing studies. For biological experiments, no data exclusions were made.
Replication	For all experiments, there are at least two independent biological repeats and multiple technical repeats in each. In all instances, all attempts at replicating the experiments produced similar results.
Randomization	For animal studies, mice were randomly assigned to treatment groups. For all other in vitro experiments, we used a common cell suspension to plate for both control and treatment groups.<br>For in vitro experiments, randomization was not applicable, as experiments were conducted using homogeneous cell populations under controlled conditions. Cells were allocated into experimental groups based on predefined treatment conditions (e.g., drug concentrations or genetic perturbations). All groups were processed in parallel using identical culture conditions to minimize variability. Given the uniform nature of the cell lines and experimental setup, there were no relevant covariates requiring additional control.
Blinding	All histo-pathological evaluations of tissues and IHC/staining-based scoring for drug toxicity studies were carried out in a blinded manner by two independent pathologists.<br>or all other experiments, blinding was not performed because data collection and quantification were conducted using automated instruments or standardized software-based pipelines (e.g., for imaging, RNA-seq, ChIP-seq, or proteomic analyses) with minimal to no subjective manual input. As such, investigator blinding was not relevant to these experiments.

Reporting for specific materials, systems and methods

We require information from authors about some types of materials, experimental systems and methods used in many studies. Here, indicate whether each material, system or method listed is relevant to your study. If you are not sure if a list item applies to your research, read the appropriate section before selecting a response.

Materials & experimental systems

n/a	Involved in the study	
☐	☒	Antibodies
☐	☒	Eukaryotic cell lines
☒	☐	Palaeontology and archaeology
☐	☒	Animals and other organisms
☒	☐	Clinical data
☒	☐	Dual use research of concern
☒	☐	Plants

Methods

n/a	Involved in the study	
☐	☒	ChIP-seq
☒	☐	Flow cytometry
☒	☐	MRI-based neuroimaging

Antibodies

Antibodies used	For WB: p300 (1:500, Invitrogen: MA1-16608, RW168, Vendor-validated); CBP (1:500, Invitrogen: PA5-27369, , Vendor-validated); AR (1:1000, Abcam: ab133273, ER179(2), Vendor-validated); H2BK5ac (1:1000, Cell Signaling Technology: 12799S, Vendor-validated); H2BK20ac (1:1000, Cell Signaling Technology: 34156S, vendor-validated); H2BK12ac (1:1000, Abcam: ab40883, EP858Y, Vendor-validated); H2BK16ac (1:1000, Abcam: ab177427, EPR17598, Vendor-validated); H3K27ac (1:1000, Cell Signaling Technology: 8173S, D5E4, Vendor-validated); H3K18ac (1:1000, Active Motif: 39755, Vendor-validated); H3K4me1 (1:1000, Abcam: ab8895, Vendor-validated); H3K4me3 (1:1000, Active Motif: 39060, Vendor-validated); H3K27me3(1:1000, Millipore: 07-449, Vendor-validated); H2B (1:1000, Active Motif: 39210, Vendor-validated); H3 (1:1000, Cell Signaling Technology: 3638S, 96C10, Vendor-validated); H2BK120ac (1:1000, Active Motif: 39119, Vendor-validated); Myc (1:1000, Cell Signaling Technology: 9402S, Vendor-validated); KLK3/PSA (1:1000, Dako: A0562, Vendor-validated); CITED2 (1:1000, Abcam: ab108345, EPR3416(2), Vendor-validated); NKX3-1 (1:1000, Cell Signaling Technology:83700S, D2Y1A, Vendor-validated); CCND1 (1:1000, Abcam: ab16663, SP4, Vendor-validated); FOXA1 (1:1000, Thermo Fisher Scientific: PA5-27157, Vendor-validated); Vinculin (1:1000, Cell Signaling Technology: 18799S, E1E9V, Vendor-validated) ; GAPDH (1:1000, Santa Cruz Biotechnology: sc-47724, 0411, Vendor-validated); BRD2 (1:1000, Bethyl Laboratories: A700-008,BL-167-2A2, Vendor-validated); BRD3 (1:1000, Bethyl Laboratories: A302-368A, Vendor-validated); BRD4 (1:1000, Bethyl Laboratories: A700-004CF, BL-149-2H5, Vendor-validated); GSPT1 (1:1000, Proteintech: 28130-1-ap, Vendor-validated); Aiolos (1:1000, Cell Signaling Technology: 15103S, D1C1E, Vendor-validated); Ikaros (1:1000, Cell Signaling Technology: 14859S, D6N9Y, Vendor-validated), HDAC3 (1:1000, Abcam, ab137704, Vendor-validated), Goat Anti-Rabbit IgG (H + L)-HRP Conjugate (1:10000, Biorad, #1706515), ECL Peroxidase (HRP) Anti-Mouse IgG (1:10000, Cytiva,NA931-100UL).<br>For ChIP-seq: H3K27ac (1ug/million cells, Diagenode: C15410196, Vendor-validated); p300 (2ug/million cells, Abcam: ab14984, G230 / NM-11, Vendor-validated); H2BK20ac (1ug/million cells, Cell Signaling Technology: 34156S, vendor-validated); H2BK5ac (1ug/million cells, Cell Signaling Technology: 12799S, Vendor-validated); H2BK16ac (1ug/million cells, Abcam, ab177427, EPR17598, Vendor-validated); AR (2ug/million cells, Millipore: 06-680, Vendor-validated); FOXA1 (2ug/million cells, Thermo Fisher Scientific: PA5-27157, Vendor-validated); BRD4 (2ug/million cells, Diagenode: C15410337, Vendor-validated); H3K18ac (1ug/million cells, Active Motif: 39755, Vendor-validated); MED1 (2ug/million cells, Active motif: 61065, Vendor-validated); ERG (2ug/million cells, Cell Signaling Technology: 97249S, A7L1G, Vendor-validated); RNA Pol II (1ug/million cells, Active motif: 39097, 4H8, Vendor-validated); Drosophila H2AV antibody for ChIP-seq Spike-in reference (1ug/25ug chromatin, Active Motif: 61686, Vendor-validated)<br>For IHC or IF: p300 (1:250, Invitrogen: 33-7600, NM11, Vendor-validated); CBP (1:100, Invitrogen: PA5-27369, Vendor-validated); AR (1:4000, Abcam: ab133273, EPR1535(2)); Ki67 (predilute, Ventana Medical Systems: 790-4286, 30-9, Vendor-validated); H2BK20ac (1:50, Abcam: ab177430, epr859, Vendor-validated); H2BK5ac (1:250, Abcam: ab40886, Vendor-validated); CCND1 (predilute, Cell Marque: 241R-18, SP4, Vendor-validated); KRT8 (1:50, Abcam: ab53280, EP1628Y); H3K27ac (1:200, Cell Signaling Technology: 8173S, D5E4, Vendor-validated), Anti-rabbit HRP (predilute, .OmniMap, 760-4311), Anti-mouse HRP (predilute, OmniMap, 760-4310);

Validation

All antibodies used in this study are from reputed commercial vendors and have been validated by the vendors (see website). QC data is directly available from all the vendors listed above, and these antibodies have been commonly used in other publications.
p300: https://www.thermofisher.com/antibody/product/p300-Antibody-clone-RW128-Monoclonal/MA1-16608, manufacturer states: This Antibody was verified by Knockdown to ensure that the antibody binds to the antigen stated.
CCND1:
CBP: https://www.thermofisher.com/antibody/product/CBP-Antibody-Polyclonal/PA5-27369, manufacturer states: This Antibody was verified by Knockdown to ensure that the antibody binds to the antigen stated.
AR:https://www.abcam.com/en-us/products/primary-antibodies/androgen-receptor-antibody-er1792-chip-grade-ab108341, manufacturer states: Advanced validation: For certain targets, additional application-specific testing is undertaken based on the most relevant methodologies for research into these targets. This additional validation testing data demonstrates efficacy to researchers when selecting products.
H2BK5ac: https://www.cellsignal.com/products/primary-antibodies/acetyl-histone-h2b-lys5-d5h1s-xp-rabbit-mab/12799?srsltid=AfmBOoo4Z-Or_MfINXDRYU6YN5FPHUPgGwDHpa3jE6n71wAZHvc8cc8B, manufacturer states: Acetyl-Histone H2B (Lys5) (D5H1S) XP® Rabbit mAb recognizes endogenous levels of histone H2B only when acetylated at Lys5. This antibody does not cross-react with other acetylated histones.
H2BK20ac: https://www.cellsignal.com/products/primary-antibodies/acetyl-histone-h2b-lys20-d7o9w-rabbit-mab/34156, manufacturer states: Acetyl-Histone H2B (Lys20) (D7O9W) Rabbit mAb recognizes endogenous levels of histone H2B protein when acetylated at Lys20. This antibody shows very slight cross-reactivity with histone H2B acetylated at Lys12.
H2BK12ac: https://www.abcam.com/en-us/products/primary-antibodies/histone-h2b-acetyl-k12-antibody-ep858y-ab40883?srsltid=AfmBOopfHUZXcMgiLrHng2CUt59dOszNQlpxaXx5KbSYUnRbA8yq3Ilq. We validated this antibody by treating the cells with p300/CBP degraders and confirmed the loss of signal by wb.
H2BK16ac: https://www.abcam.com/en-us/products/primary-antibodies/histone-h2b-acetyl-k16-antibody-epr17598-chip-grade-ab177427#drawerView=highlights, manufacturer states: Advanced validation: For certain targets, additional application-specific testing is undertaken based on the most relevant methodologies for research into these targets. This additional validation testing data demonstrates efficacy to researchers when selecting products.
h3k27ac: https://www.cellsignal.com/products/primary-antibodies/acetyl-histone-h3-lys27-d5e4-xp-rabbit-mab/8173?srsltid=AfmBOorhibRWiQMGztdR2Yz3l9Nwia6wXCx0qi48PpbsDuPSyNE_pe_q, manufacturer states: Acetyl-Histone H3 (Lys27) (D5E4) XP® Rabbit mAb recognizes endogenous levels of histone H3 protein only when acetylated at Lys27. This antibody does not cross react with histone H3 acetylated at Lys9, 14, 18, 23, or 56. This antibody shows some cross-reactivity with acetyl-histone H2B lysine 5.
H3K18ac: https://www.activemotif.com/catalog/details/39755/histone-h3-acetyl-lys18-antibody-pab-3, manufacturer states: Applications Validated by Active Motif: ChIP: 10 µg per ChIP, ChIP-Seq: 4 µg each, ICC/IF: 1 µg/ml dilution, WB: 0.5 - 2 µg/ml dilution, DB: 0.1 - 0.5 µg/ml dilution
H3K4me1, https://www.abcam.com/en-us/products/primary-antibodies/histone-h3-mono-methyl-k4-antibody-chip-grade-ab8895?srsltid=AfmBOopgxryvWLRLafl8oXO_q5ZPjnd6RIXp74wxQUtkXG1ftMeoOQGN, the manufacturer states: Specific for Mono Methyl-K4. Does not Di Methyl-K4, Tri Methyl-K4 or Methyl-K9, Cited in over 1,100 publications, Trusted by researchers since 2002.
H3K4me3:https://www.activemotif.com/catalog/details/39159/histone-h3-trimethyl-lys4-antibody-pab, manufacturer states: Applications Validated by Active Motif: ChIP: 3 - 5 µl per ChIP, ChIP-Seq: 3 µl each, ICC/IF: 1:500 - 1:1,000 dilution, WB: 1:500 - 1:2,000 dilution, CUT&Tag: 1 µl per 50 µl reaction*, CUT&RUN: 1 µl per 50 µl reaction
H3K27me3: https://www.sigmaaldrich.com/US/en/product/mm/07449?srsltid=AfmBOorAvb EymMMPOF1qMbXr5HuR6mumQQRq-1Qb7NlrX04VESBTeJW, manufacturer states: Anti-trimethyl-Histone H3 (Lys27), also known as Anti-H3K27me3, is a highly published Rabbit Polyclonal Antibody. This protein A purified antibody is dot blot tested for trimethylated lysine 27 specificity and validated in WB, ICC, IP.
H2B: https://www.activemotif.com/catalog/details/39210/histone-h2b-antibody-pab, manufacturer states: Applications Validated by Active Motif: WB: 2 µg/ml dilution
H3: https://www.cellsignal.com/products/primary-antibodies/histone-h3-96c10-mouse-mab/3638?srsltid=AfmBOoqW82QOeXZGI5TVOfZNMZLO8lDnHkkR3yDERs8X9-kqtrnYd1jq, manufacturer states: Histone H3 (96C10) Mouse mAb detects endogenous levels of total Histone H3 protein, including isoforms H3.1, H3.2, and H3.3. The antibody does not cross-react with other histone proteins, including the Histone H3 variant CENP-A.
H2BK120ac: https://www.activemotif.com/catalog/details/39119/histone-h2b-acetyl-lys120-antibody-pab, manufacturer states: Applications Validated by Active Motif: ChIP: 10 µl per ChIP, ChIP-Seq: 5 µl each, WB*: 1:1,000 - 1:2,000 dilution, IF: 1:500 dilution.
Myc: https://www.cellsignal.com/products/primary-antibodies/c-myc-antibody/9402, manufacturer states: c-Myc Antibody detects endogenous levels of total c-Myc protein. This antibody is not recommended for detection of Myc-tagged fusion proteins (use Cell Signaling Technology cat. #2276 or #2278).
KLK3/PSA: https://www.citeab.com/antibodies/3382929-a0562-prostate-specific-antigen-psa, from citeab website: A polyclonal rabbit antibody, supplied by Dako, raised against Prostate-specific antigen (Human), cited in 99 publications, with 20 published images. Applications used include WB, IHC, ICC, ICC-IF, and 2 others.
CITED2: https://www.abcam.com/en-us/products/primary-antibodies/cited2-antibody-epr34162-ab108345?srsltid=AfmBOopYzwPzu1ZLHz2jH8km5rPGZNUMnviURy4BdRU3u-IVluOg92YK. We validated this antibody by using CITED2 siRNA and confirm the loss of signal by wb.
NKX3-1: https://www.cellsignal.com/products/primary-antibodies/nkx3-1-d2y1a-xp-rabbit-mab/83700, manufacturer states: NKX3.1 (D2Y1A) XP® Rabbit mAb recognizes endogenous levels of total NKX3.1 protein.
CCND1: https://www.abcam.com/en-us/products/primary-antibodies/cyclin-d1-antibody-sp4-ab16663?srsltid=AfmBOoryNSb9tl0ysvpoW6uCU1_v2iJV6hrFVJKaUnloONhtjMYGeeuq#drawerView=highlights. Manufacturer claims this is a KO validated antibody.
FOXA1: https://www.thermofisher.com/antibody/product/FOXA1-Antibody-Polyclonal/PA5-27157. Manufacturer states: This Antibody was verified by Relative expression to ensure that the antibody binds to the antigen stated and Antibody specificity was demonstrated by detection of differential basal expression of the target across cell lines owing to their inherent genetic constitution. Enrichment of FOXA1 active binding region was observed in LNCaP cells in comparison to SW480 cells using Anti- FOXA1 Polyclonal Antibody (Product # PA5-27157) in chromatin immunoprecipitation.
Vinculin: https://www.cellsignal.com/products/antibody-conjugates/vinculin-e1e9v-xp-rabbit-mab-hrp-conjugate/18799?srsltid=AfmBOorV6mhoep8U_KdKI2_5v1XMGXsPElysTEwwEc-yalgv9CLE1v4A, manufacturer states: Vinculin (E1E9V) XP® Rabbit mAb (HRP Conjugate) recognizes endogenous levels of total vinculin protein. This antibody also reacts with metavinculin, a 145 kDa splice variant of vinculin.
GAPDH: https://www.scbt.com/p/gapdh-antibody-0411?srsltid=AfmBOopy1PEuNKckOJxVvbiiHZpv4mN-JksMdtk00a-8UOTvaEI7CZT_.

This antibody has been cited in more than 3300 papers.
BRD2: https://www.fortislife.com/products/primary-antibodies/rabbit-anti-brd2-recombinant-monoclonal-antibody-bl-167-2a2/BETHYL-A700-008. Manufacturer states: This antibody has been validated by Pillar 1: Independent Antibodies, Pillar 2: Complementary Assays, Pillar 4: Biological Characteristics.
BRD3: https://www.fortislife.com/products/primary-antibodies/rabbit-anti-brd3-antibody/BETHYL-A302-368?selected=A302-368A. Manufacturer states: this antibody has been validated to meet their strict performance standards. And this antibody has been cited in 52 papers.
BRD4: https://www.fortislife.com/products/primary-antibodies/rabbit-anti-brd4-recombinant-monoclonal-antibody-bl-149-2h5/BETHYL-A700-004?selected=A700-004CF, manufacturer states that this antibody has been validated by Pillar 1: Independent Antibodies, Pillar 2: Complementary Assays. We also validated this antibody by treating the cells with BRD4 PROTAC degader and confirm the loss wb signal.
GSPT1: https://www.ptglab.com/products/GSPT1-Antibody-28130-1-AP.htm?srsltid=AfmBOopde6wBFxuVMSRA76-1gLZMH5u_IlD0JdnkwFjr6XZ8G91V5zur, manufacturer states: 28130-1-AP targets eRF3a/GSPT1 in WB, IHC, IF/ICC, ELISA applications and shows reactivity with human, mouse, rat samples.
Aiolos: https://www.cellsignal.com/products/primary-antibodies/aiolos-d1c1e-rabbit-mab/15103, manufacturer states: Aiolos (D1C1E) Rabbit mAb recognizes endogenous levels of total Aiolos protein.
Ikaros: https://www.cellsignal.com/products/primary-antibodies/ikaros-d6n9y-rabbit-mab/14859, manufacturer states: Ikaros (D6N9Y) Rabbit mAb recognizes endogenous levels of total Ikaros protein.
HDAC3: https://www.citeab.com/antibodies/762917-ab137704-anti-hdac3-antibody. This antibody has been cited in 14 publications. We have validated this antibody by using siHDAC3.
MED1: https://www.activemotif.com/catalog/details/61065/med1-antibody-pab. We have validated this antibody by ChIP-seq and compared the MED1 peaks with a published dataset in the same cell line.
ERG: https://www.cellsignal.com/products/primary-antibodies/erg-a7l1g-rabbit-mab/97249. Manufacturer states: ERG (A7L1G) Rabbit mAb recognizes endogenous levels of total ERG protein. Based on sequence identity, this antibody should detect isoforms ERG1, ERG2 and ERG3. This antibody does not cross-react with Fli1. Non-specific staining was observed in human stomach epithelium by immunohistochemistry. This antibody is not recommended for immunohistochemical analysis of mouse tissues. We also validated this antibody by using siERG.
RNA Pol2: https://www.activemotif.com/catalog/details/39097/rna-pol-ii-antibody-mab. Manufacturer states: Applications Validated by Active Motif: ChIP: 10 µl per ChIP, ChIP-Seq: 6-20 µl each, WB: 1:2,000 - 1:5,000 dilution.
Drosophila H2AV antibody: https://www.activemotif.com/catalog/1091/chip-normalization. This antibody is a Spike-in reference antibody for ChIP-seq, which has been validated by Active motif.
p300: https://www.thermofisher.com/antibody/product/p300-Antibody-clone-NM-11-Monoclonal/33-7600. Manufacturer states: This Antibody was verified by Knockdown to ensure that the antibody binds to the antigen stated. Antibody specificity was demonstrated by siRNA mediated knockdown of target protein. HCT 116 cells were transfected with p300 siRNA and decrease in signal intensity was observed in ICC application using Anti-p300 Monoclonal Antibody (NM-11) (Product # 33-7600).
AR: https://www.abcam.com/en-us/products/primary-antibodies/androgen-receptor-antibody-epr15352-ab133273. We validated this antibody by siAR.
kI67: https://elabdoc-prod.roche.com/eLD/web/global/en/products/RTD000792?searchTerm=&catalog=ProductCatalog&orderBy=Relevance&fromResults=true. Manufacturer states: CONFIRM anti-Ki-67 (30-9) Rabbit Monoclonal Primary Antibody is intended for laboratory use in the qualitative immunohistochemical detection of Ki-67 protein by light microscopy in sections of formalin-fixed, paraffin-embedded tissue stained on a BenchMark IHC/ISH instrument. This product should be interpreted by a qualified pathologist in conjunction with histological examination, relevant clinical information, and proper controls. This antibody is intended for in vitro diagnostic (IVD) use.
CCND1: https://www.cellmarque.com/antibodies/CM/63/Cyclin-D1_SP4. This antibody has been validated by manufacturer and applied for the diagnosis of mantle cell lymphomas.
CK8: https://www.abcam.com/en-us/products/primary-antibodies/cytokeratin-8-antibody-ep1628y-cytoskeleton-marker-ab53280. Manufacturer states that this antibody has been validated by KO.
H3K27ac: https://www.abcam.com/en-us/products/primary-antibodies/histone-h3-acetyl-k27-antibody-chip-grade-ab4729?srsltid=AfmBOorViyxTaMVZTHNGhPvI93j11sGtG3b68nvTpWY_1ak3vjQQd9Xl. Manufacturer states: This antibody is tried and trusted by researchers since 2004. There are 2118 citations of this antibody.

Eukaryotic cell lines

Policy information about cell lines and Sex and Gender in Research

Cell line source(s)	Most cell lines were originally obtained from ATCC, DSMZ, ECACC, Lonza, or internal stock. CWR-R1 cells and a series of enzalutamide-resistant prostate cancer cell lines (LNCaP_Parental, LNCaP_EnzR) were provided by D. Vander Griend (University of Illinois at Chicago). Cells were grown in media conditions prescribed by ATCC, DSMZ, or ECACC.
Authentication	All cell lines were genotyped every month at the University of Michigan Sequencing Core using Profiler Plus (Applied Biosystems) and compared with corresponding short tandem repeat (STR) profiles in the ATCC database to authenticate their identity in culture between passages and experiments.
Mycoplasma contamination	All cell lines were biweekly tested to be free of mycoplasma contamination.
Commonly misidentified lines (See ICLAC register)	None

Animals and other research organisms

Policy information about studies involving animals; ARRIVE guidelines recommended for reporting animal research, and Sex and Gender in Research

Laboratory animals	Efficacy studies: 4-6 week old male CB17 severe combined immunodeficiency (SCID) mice were procured from the University of Michigan breeding colony. Pharmacokinetics study: 9-11 week old CD-1 male mice, 9-12 week humanized cereblon C57BL/6 and 12 week-old male CD rats were used. All mice were maintained under the conditions of pathogen-free, 12 hours light/12 hours dark cycle, temperatures of 18-23°C, and 40-60% humidity.
Wild animals	No wild animals were used in the study.
Reporting on sex	Male animals were used since prostate cancer is specific to males.
Field-collected samples	No field collected samples were used in the study.
Ethics oversight	Institutional Review Board and Institutional Animal Care and Use Committee at the University of Michigan ensures that the highest animal welfare standards are maintained along with the conduct of accurate, valid scientific research through the supervision, coordination, training, guidance, and review of every project proposed to include the use of vertebrate animals at the University of Michigan. Protocl number PRO0001291

Note that full information on the approval of the study protocol must also be provided in the manuscript.

Plants

Seed stocks	Not applicable
Novel plant genotypes	Not applicable
Authentication	Not applicable

ChIP-seq

Data deposition

☒ Confirm that both raw and final processed data have been deposited in a public database such as GEO.

☒ Confirm that you have deposited or provided access to graph files (e.g. BED files) for the called peaks.

Data access links<br>*May remain private before publication.*	All sequencing data generated in this study have been deposited in the following National Center for Biotechnology Information Gene Expression Omnibus (NCBI GEO) repository: GSE255134.
Files in database submission	These ChIP-seq Fastq files are submitted to the GEO:<br>ChIP-seq: VCaP DMSO_H3K27ac<br>VCaP CBPD_4hrs_H3K27ac<br>VCaP GNE_4hrs_H3K27ac<br>VCaP CCS_4h_H3K27ac<br>VCaP_DMSO_AR<br>VCaP_CBPD_4h_AR<br>VCaP_GNE_4h_AR<br>VCaP_DMSO_FOXA1<br>VCaP_CBPD_4h_FOXA1<br>VCaP_GNE_4h_FOXA1<br>VCaP_DMSO_H2BK5ac<br>VCaP_CBPD_4hrs_H2BK5ac<br>VCaP_GNE_4hrs_H2BK5ac<br>VCaP_DMSO_H2BK20ac<br>VCaP_CBPD_4hrs_H2BK20ac<br>VCaP_GNE_4hrs_H2BK20ac<br>VCaP_P300<br>VCaP_DMSO_BRD4<br>VCaP_MED1_AM<br>VcaP_DMSO_Pol2<br>VcaP_CBPD_4h_Pol2

VcaP_GNE_4h_Pol2
VCaP_DMSO_H3K18ac
VCaP_CBPD_4hrs_H3K18ac
VCaP_GNE_4hrs_H3K18ac
VCap_Prathiba_May_HC7C2DRX3
DMSO_AR SI_28420
DMSO_FOXA1 SI_28421
AU15330_AR SI_28422
AU15330_FOXA1 SI_28423
VCaP_H2BK20ac_CST
DU145_H2BK20ac_CST
PC3_H2BK20ac_CST
VCaP_H2BK16ac_abcam
DU145_H2BK16ac_abcam
PC3_H2BK16ac_abcam
VCaP_DMSO_BRD4
VCaP_CBPD_4h_BRD4

Genome browser session (e.g. UCSC)

N/A

Methodology

Replicates

Multiple biological as well as technical replicates are included.

Sequencing depth

ATAC-seq: Sequenced to 65-70M total reads, paired-end mode, 125bp read lengths. Over 97% of uniquely mapped reads.
ChIP-seq: Sequenced to 50-70M total reads, paired-end mode, 125bp read lengths. Over 97% of uniquely mapped reads.
RNA-seq: Sequenced to 30-40M total reads, paired-end mode, 125bp read lengths. Over 97% of uniquely mapped reads.
EU-seq: Sequenced to 25-30M total reads, paired-end mode, 125bp read lengths. Over 97% of uniquely mapped reads.

Antibodies

For ChIP-seq, the following antibodies were used: H3K27ac (Diagenode: C15410196); p300 (Abcam: ab14984); H2BK20ac (Cell Signaling Technology: 34156S); H2BK5ac (Cell Signaling Technology: 12799S); H2BK16ac (Abcam, ab177427); AR (Millipore: 06-680); FOXA1 (Thermo Fisher Scientific: PA5-27157); BRD4 (Diagenode: C15410337); H3K18ac (Active Motif: 39755); MED1 (Active motif: 61065); ERG (Cell Signaling Technology: 97249S); RNA Pol II (Active motif: 39097), Drosophila H2AV antibody for ChIP-seq Spike-in reference (Active Motif: 61686)

Peak calling parameters

MACS2 was used for peak calling with narrowpeak setting for narrow peaks and a second set of parameters for histone peaks (eg H3K27Ac, --broad -B --cutoff-analysis --broad-cutoff 0.05 --max-gap 500).

Data quality

FastQC was used to quality check the raw sequencing data using standard metrics and default thresholds.

Software

ChIP-seq data analysis started with trimming using Trimmomatic version 0.39 (settings TruSeq3-PE-2.fa:2:30:10, minlen 50) [Trim]. BWA was used to align reads to hg38 (GRCh38) human genome reference ("bwa mem" command with options -5SP -T0, version 0.7.17-r1198-dirty) [bwa]. Alignments were filtered using samtools (quality score cutoff of 20) and picard MarkDuplicates (removed duplicates) [picard, samtools]. MACS2 was used for peak calling with narrowpeak setting for narrow peaks and a second set of parameters for histone peaks (eg H3K27Ac, --broad -B --cutoff-analysis --broad-cutoff 0.05 --max-gap 500) [MACS2]. In addition, bedtools was used to remove blacklisted regions of the genome from the peaks list (Encode's exclusion list ENCFF356LFX.bed) [bedtools, encode]. UCSC's tool wigtoBigwig was used for conversion to bigwig formats [wigtoBigwig].
