# Supplementary material for: Targeting histone H2B acetylated enhanceosomes via p300/CBP degradation in prostate cancer
Source: Nat Genet. 2025 Oct 3;57(10):2468–81. doi: 10.1038/s41588-025-02336-6 (PMC12513837; doi:10.1038/s41588-025-02336-6)

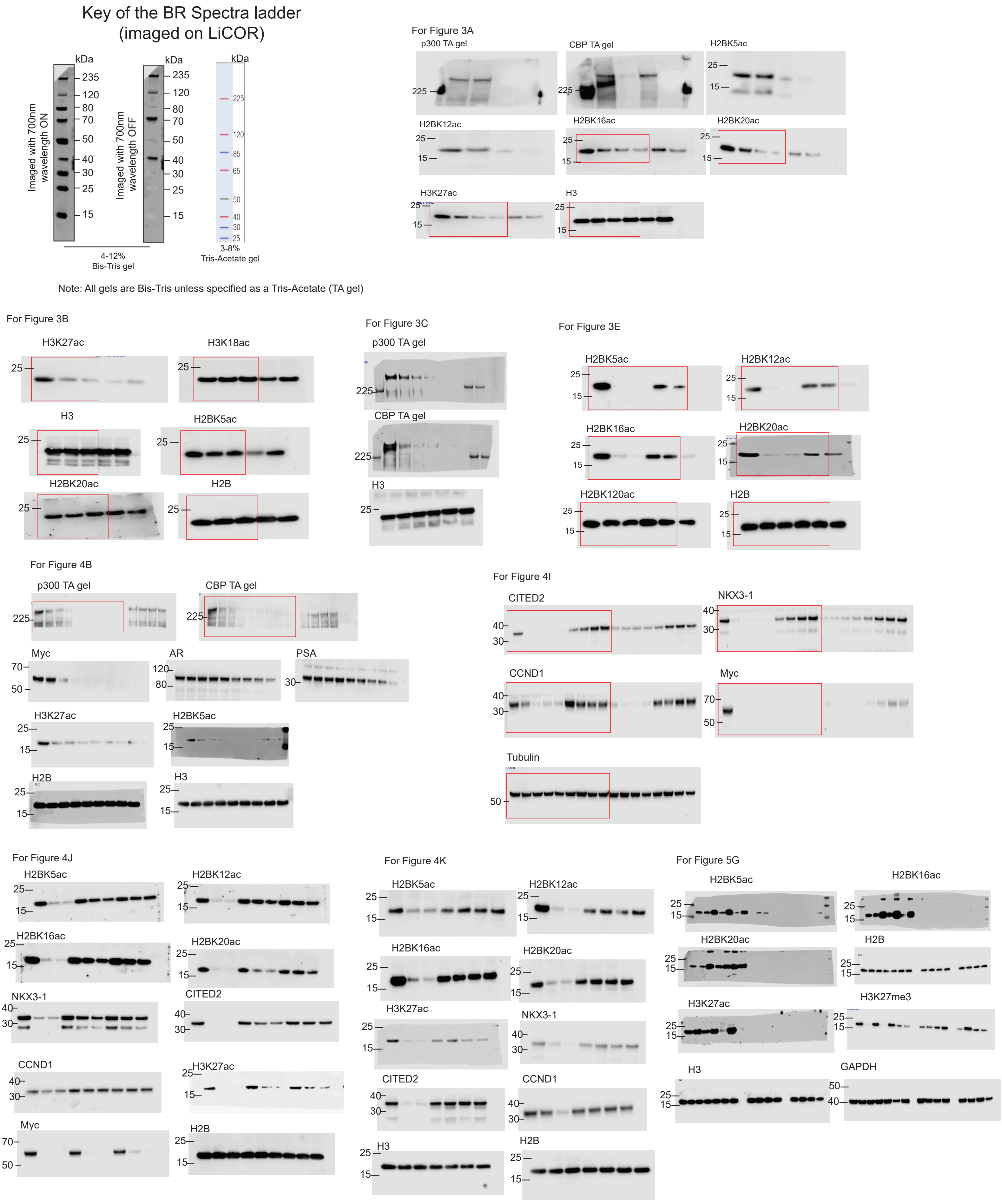
Key of the BR Spectra ladder (imaged on LiCOR)
Imaged with 700nm wavelength ON
Imaged with 700nm wavelength OFF
kDa
235
120
80
70
50
40
30
25
15
225
120
85
65
50
40
30
25
4-12% Bis-Tris gel
3-8% Tris-Acetate gel
Note: All gels are Bis-Tris unless specified as a Tris-Acetate (TA gel)
For Figure 3A
p300 TA gel
CBP TA gel
H2BK5ac
H2BK12ac
H2BK16ac
H2BK20ac
H3K27ac
H3
For Figure 3B
H3K27ac
H3K18ac
H3
H2BK5ac
H2BK20ac
H2B
For Figure 3C
p300 TA gel
CBP TA gel
H3
For Figure 3E
H2BK5ac
H2BK12ac
H2BK16ac
H2BK20ac
H2BK120ac
H2B
For Figure 4B
p300 TA gel
CBP TA gel
Myc
AR
PSA
H3K27ac
H2BK5ac
H2B
H3
For Figure 4I
CITED2
NKX3-1
CCND1
Myc
Tubulin
For Figure 4J
H2BK5ac
H2BK12ac
H2BK16ac
H2BK20ac
NKX3-1
CITED2
CCND1
H3K27ac
Myc
H2B
For Figure 4K
H2BK5ac
H2BK12ac
H2BK16ac
H2BK20ac
H3K27ac
NKX3-1
CITED2
CCND1
H3
H2B
For Figure 5G
H2BK5ac
H2BK16ac
H2BK20ac
H2B
H3K27ac
H3K27me3
H3
GAPDH

For ED Fig. 1A

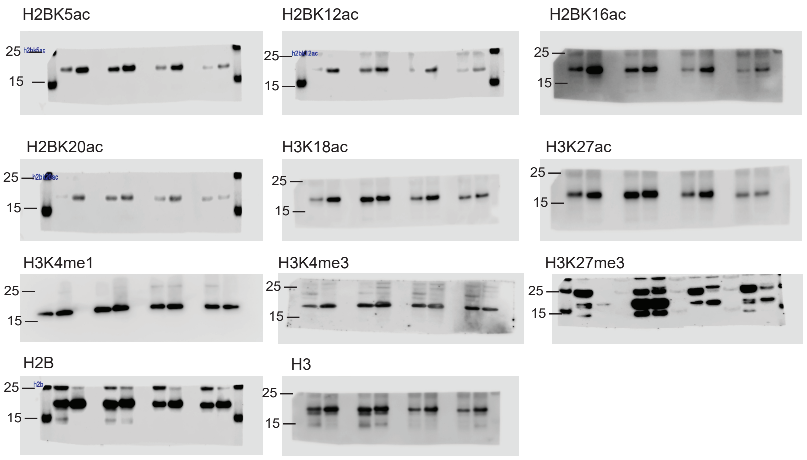

For ED Fig. 3A

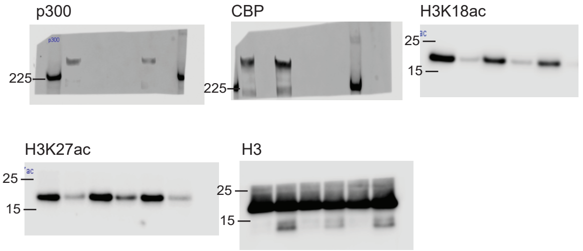

For ED Fig. 3D

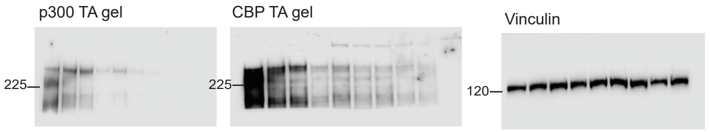

For ED Fig. 3E

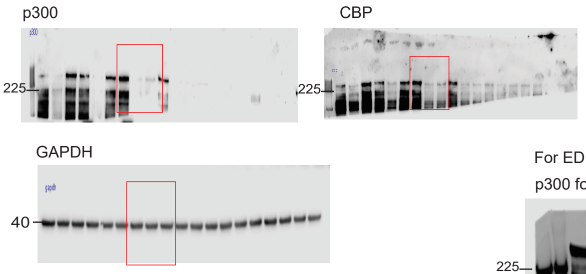

For ED Fig. 3F

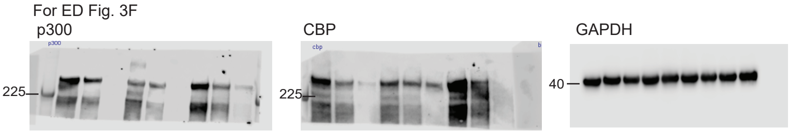

For ED Fig. 3G

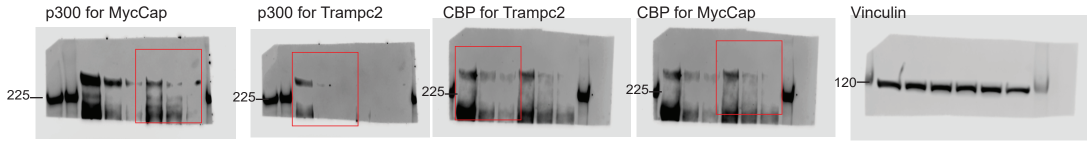

For ED Fig. 3J

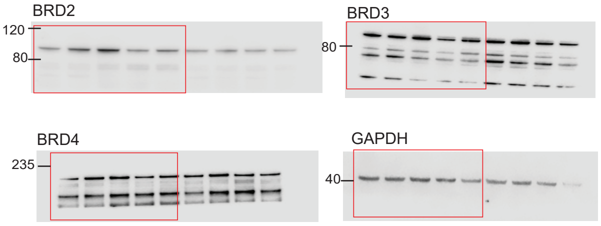

For ED Fig. 3K

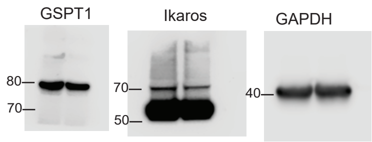

For ED Fig. 3L

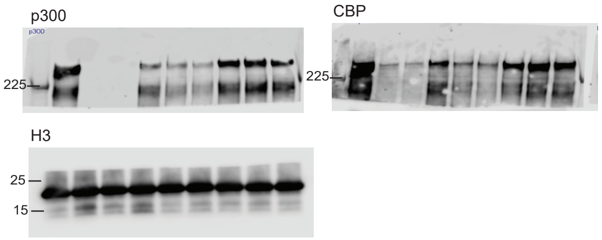

For ED Fig. 3M

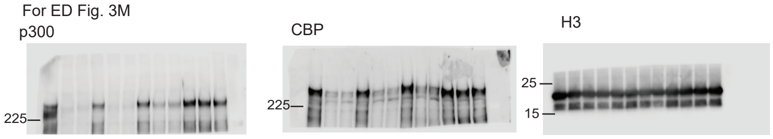

For ED Fig. 3O

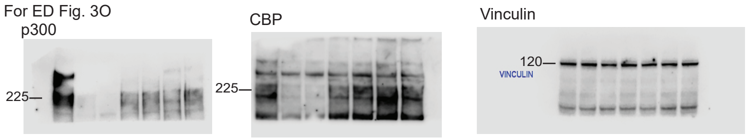

For ED Fig. 3P

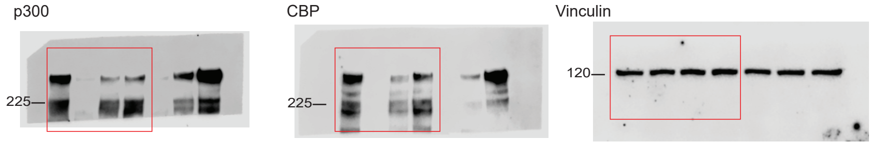

For ED Fig. 4A

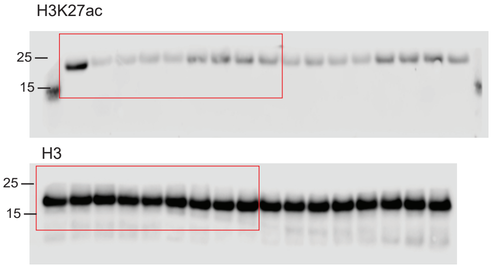

For ED Fig. 4B

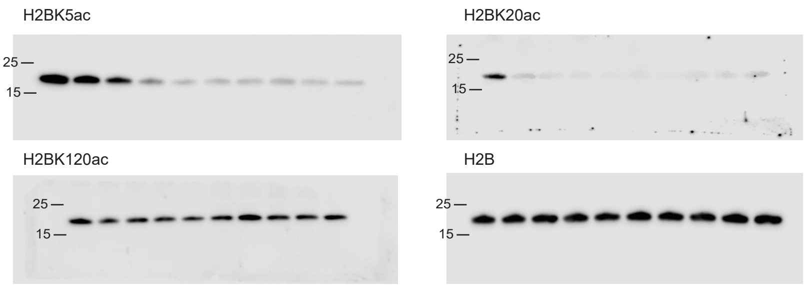

For ED Fig. 4F

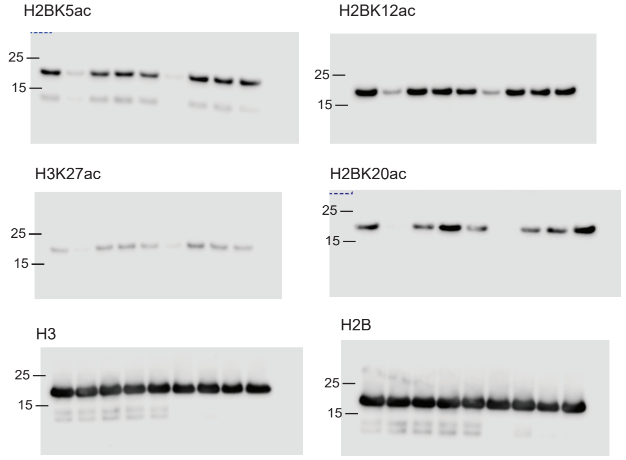

For ED Fig. 5K

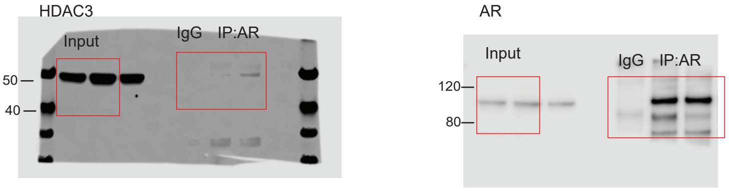

For ED Fig. 6E

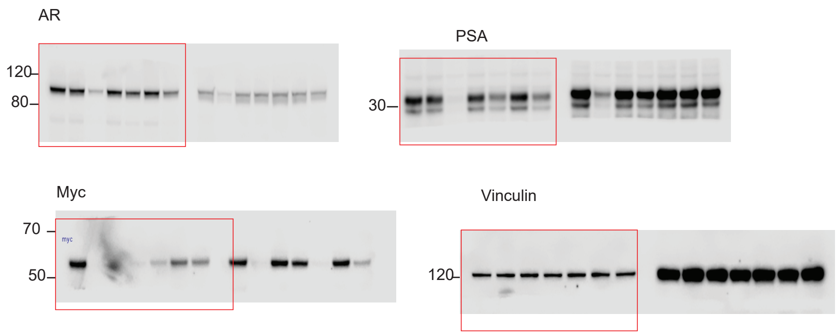

For ED Fig. 7F

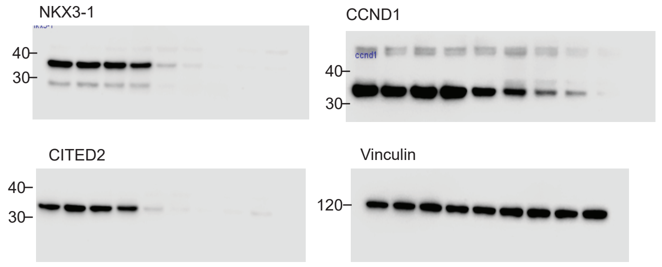

For ED Fig. 7G

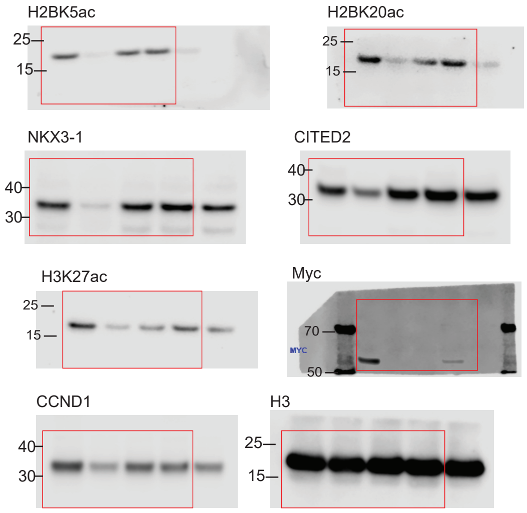

For ED Fig. 7J

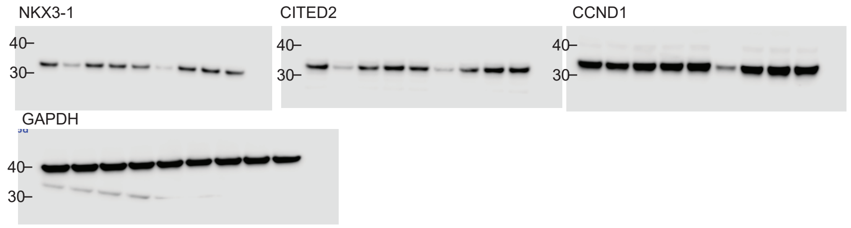

For ED Fig. 8M

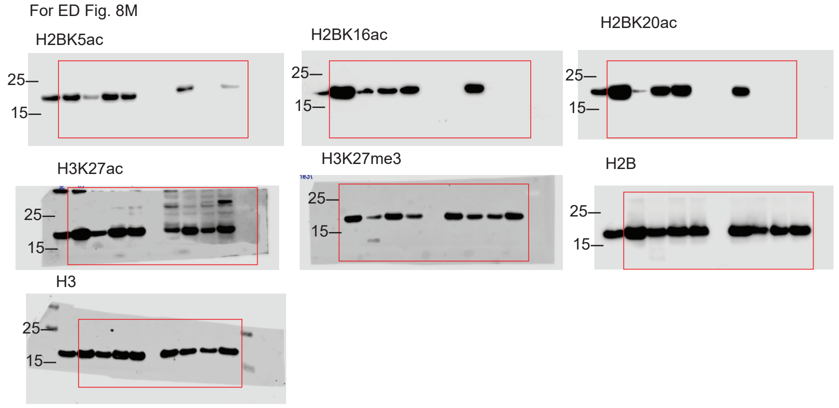

For ED Fig. 10L

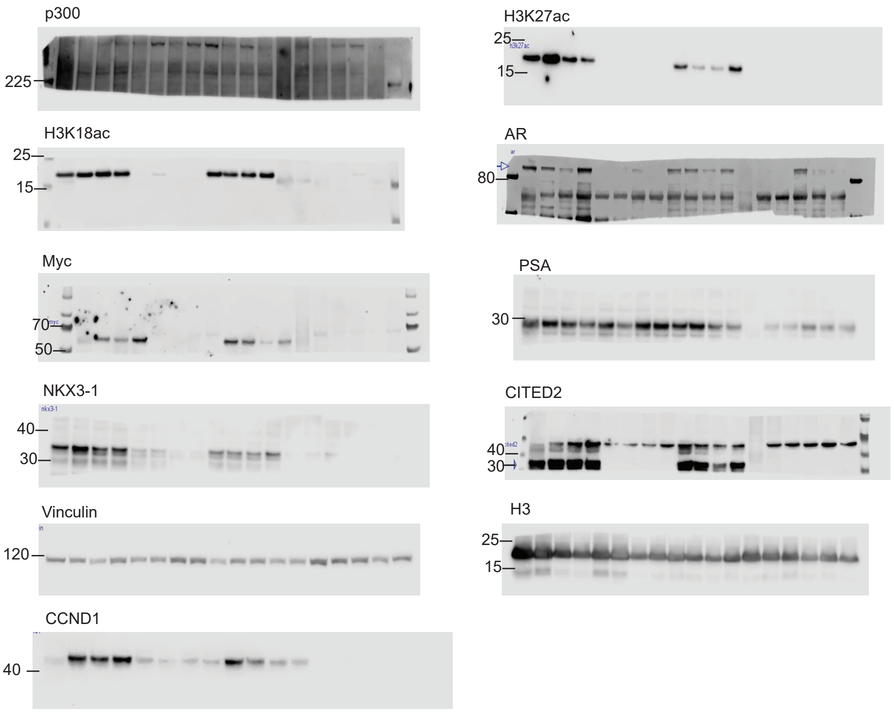

Supplement: Supplementary file 19 — Uncropped western blots for all figures and Extended Data figures. [file 41588_2025_2336_MOESM19_ESM.pdf]
